# Macroclimatic conditions as main drivers for symbiotic association patterns in lecideoid lichens along the Transantarctic Mountains, Ross Sea region, Antarctica

**DOI:** 10.1038/s41598-021-02940-6

**Published:** 2021-12-06

**Authors:** Monika Wagner, Georg Brunauer, Arne C. Bathke, S. Craig Cary, Roman Fuchs, Leopoldo G. Sancho, Roman Türk, Ulrike Ruprecht

**Affiliations:** 1 Department of Biosciences, Paris Lodron Universität Salzburg, Salzburg, Austria; 2 Department of Mathematics, Paris Lodron Universität Salzburg, Salzburg, Austria; 3 School of Science, The University of Waikato, Hamilton, New Zealand; 4 The International Centre for Terrestrial Antarctic Research, School of Science, The University of Waikato, Hamilton, New Zealand; 5 Botany Unit, Facultad de Farmacia, Universidad Complutense de Madrid, Madrid, Spain

**Keywords:** Biodiversity, Biogeography, Climate-change ecology, Community ecology, Ecological modelling, Ecological networks, Molecular ecology

## Abstract

Lecideoid lichens as dominant vegetation-forming organisms in the climatically harsh areas of the southern part of continental Antarctica show clear preferences in relation to environmental conditions (i.e. macroclimate). 306 lichen samples were included in the study, collected along the Ross Sea coast (78°S–85.5°S) at six climatically different sites. The species compositions as well as the associations of their two dominant symbiotic partners (myco- and photobiont) were set in context with environmental conditions along the latitudinal gradient. Diversity values were nonlinear with respect to latitude, with the highest alpha diversity in the milder areas of the McMurdo Dry Valleys (78°S) and the most southern areas (Durham Point, 85.5°S; Garden Spur, 84.5°S), and lowest in the especially arid and cold Darwin Area (~ 79.8°S). Furthermore, the specificity of mycobiont species towards their photobionts decreased under more severe climate conditions. The generalist lichen species *Lecanora fuscobrunnea* and *Lecidea cancriformis* were present in almost all habitats, but were dominant in climatically extreme areas. *Carbonea vorticosa, Lecidella greenii* and *Rhizoplaca macleanii* were confined to milder areas. In summary, the macroclimate is considered to be the main driver of species distribution, making certain species useful as bioindicators of climate conditions and, consequently, for assessing the consequences of climate change.

## Introduction

**Figure 1:**
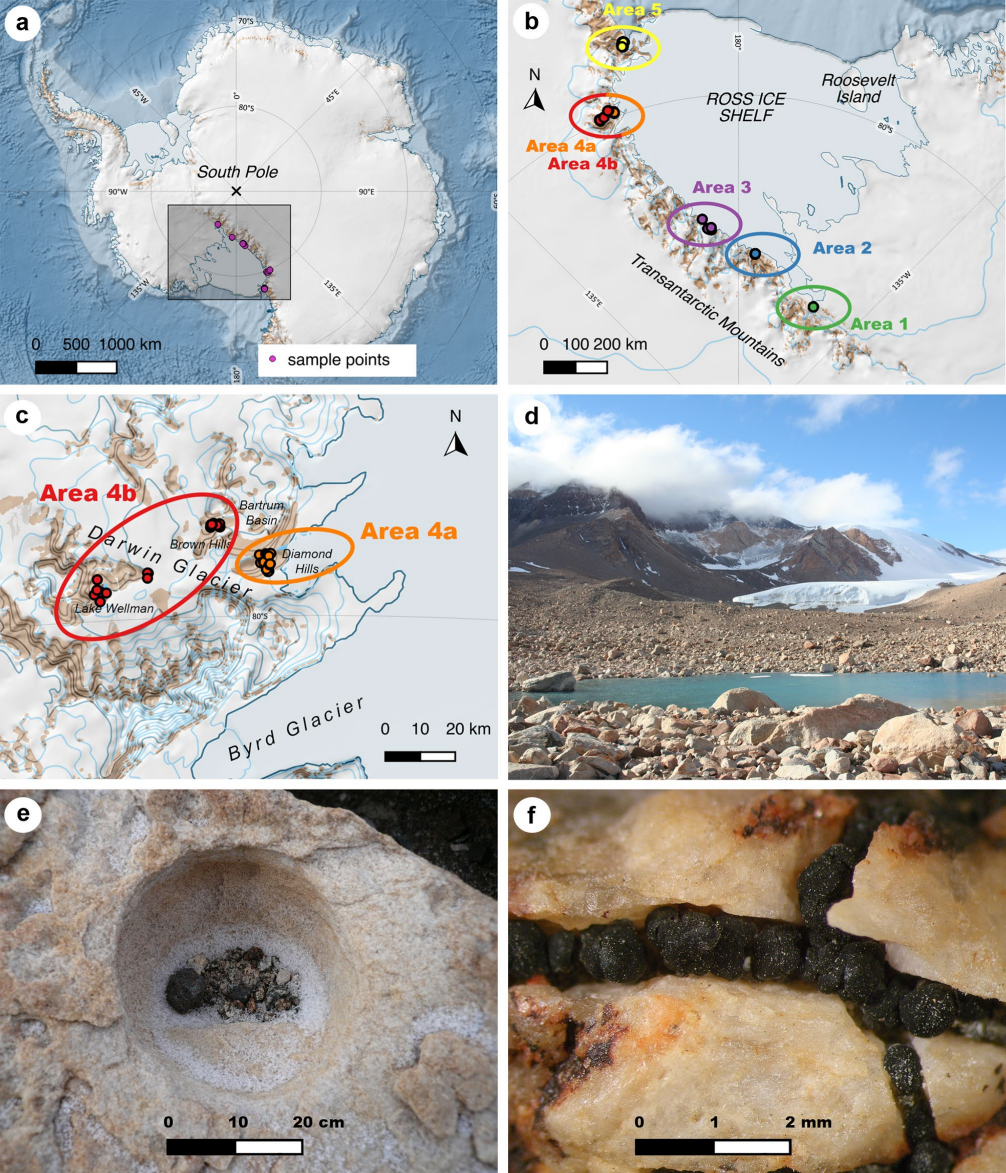
Location of the sample points and lichen habitats. (**a**) Antarctic continent, investigated area marked with rectangle, (**b**) location of the six different areas defined in the study, (**c**) differentiation of area 4 in subareas 4a and 4b, (**d**) Batrum Basin, (**e**) microhabitat with crustose lichens at Lake Wellman, (**f**) chasmolithic growth of *Lecidea cancriformis*. Maps of (**a**), (**b**) and (**c**) were created with QGIS 3.12 (http://www.qgis.org/) and are based on the dataset Quantarctica^18^.

Polar deserts of the southernmost areas in continental Antarctica are characterized by exceptionally hostile climatic conditions, such as particularly low temperatures and high aridity^1–3^.Terrestrial life is restricted to ice-free areas, which, apart from a few nunataks, are mainly located along the Transantarctic Mountains forming the west coast of the Ross Sea and Ross Ice Shelf^4^. Because of these special conditions, terrestrial life is rare and can only be found in small areas protected from extreme environmental influences, such as abrasion from windblown particles or high solar radiation, the so-called microhabitats^5,6^. They are characterized by sheltered areas in rock crevices or small cavities shielded from the wind and sun, that allow life on a small scale in an otherwise hostile environment. The rock surface is often highly weathered, which results in a higher water retention capacity, providing the most needed life source for the organisms to survive^7,8^. The only moisture available to rock-dwelling organisms is provided by clouds, fog, dew, sparse precipitation and melting snow^9,10^. Additionally, the aspect of the slopes, ridges and depressions as well as the wind regime have an important impact by creating different surface temperatures in small areas^11,12^. Macroclimate, especially mean annual temperature, seems to be the main driver of biodiversity and productivity along the latitudinal gradient in Antarctica, but the presence or absence of lichen communities in continental Antarctica depends on suitable microclimatic conditions^13^. However, microhabitats are influenced by both macroclimate and geography, and their life-supporting properties therefore vary along environmental gradients reflected in changing diversity levels and biogeography of Antarctic terrestrial biota^1,14–17^ (Fig. 1).

The terrestrial vegetation along the Ross Sea coast (extending from 72°S, Cape Hallett, to 85.5°S, Queen Maud Mountains) is entirely composed of cryptogrammic organisms and dominated by lichens and mosses^8,17,19^. Remarkably, the biodiversity of these organisms does not decrease evenly along the latitudinal gradient as one might expect. In fact, the lowest species diversity was recorded at about 79°S at Diamond Hill (Darwin Area), which has by far the harshest climate conditions, resulting in the lowest humidity^8^. However, nonlinear climatic conditions along gradients caused by additional factors, e.g. special wind systems, can be detected effectively with biological systems that act as bioindicators^10,20–22^. Additionally, they not only enable the survey of the current state but can also reliably indicate changes in environmental conditions^23^. Due to the structure and diversity of communities, the abundance and distribution of species as well as processes varying along environmental gradients are therefore powerful long-term and large-scale study systems to estimate the consequences of climate change on ecosystems^23^.

The most abundant vegetation-forming organisms in these areas are lichens, in most of the cases with a crustose thallus fused to the rocky surface or deeply embedded in crevices^6,8,24–26^. The poikilohydric lifestyle of lichens enables them to survive the harsh climate conditions and the long periods without water and/or light in a dormant state^27^. The symbiotic lifeform of lichens consists of two dominant symbiotic partners: the mycobiont (fungus) and the photosynthetic partner (green algae and/or cyanobacteria: photobiont) and additional associated fungal, algal and bacterial communities forming the holobiome lichen thallus^28–32^. Therefore, lichens constitute an excellent model for analyzing multi-species associations in one unit to reveal phylogenetic and ecological responses for symbiotic associations.

Many analyses focused on myco-/photobiont associations have demonstrated that they react sensitively to even small environmental gradients^10,33^. These results allow the conclusion that mycobionts which are less specialized to specific locations and are able to use a broader range of photobionts, such as the widespread species *Lecidea cancriformis* in continental Antarctica^10,16^, are less vulnerable to climate changes. Low photobiont specificity may improve the performance of the lichen symbiosis, e.g. by increasing the adaptive potential to new environmental conditions, and widening the geographical range via ecological niche shifts^34–36^. On the other hand, high levels of photobiont specificity are expected under conditions where ecological factors, especially (macro-) climate and/or substrate (e.g. calcareous or siliceous rock), exert a strong selective influence on lichen performance ^36–38^. Additionally, genetic identity can play a significant role in shaping myco-/photobiont associations along gradients ^39^ or may also lead to turnover zones, suggesting that photobionts are replaced by others as environmental conditions change ^40^. An influence on the selection of *Trebouxia* OTUs due to temperature combined with water availability was suggested in several studies as a key factor of photobiont selection of lichens in Antarctica ^10,41^. Due to the sensitive response of lichen communities to climatic change with modified species compositions and reduced diversity ^20,42–44^, lichen growth, abundance and diversity are expected to be negatively affected by climatic changes ^44^. Consequently, lichens represent excellent bioindicators because of their sensitive responses to environmental changes ^20,45–47^, and especially abundant and cosmopolitan species serve as a valuable model system to record diversity and composition along climatic gradients worldwide.

The current study focuses on the association patterns of the two main symbionts (myco- and photobiont) of the lecideoid lichen group^48^ that is dominant along the investigated part of the latitudinal gradient (78–85°S) at the Ross Sea coast. The following objectives were addressed: (1) to assess the biodiversity and genetic identity of the symbiotic partners of lecideoid lichens using phylogenic methods; (2) to investigate how the variability of myco-/photobiont associations is related to environmental variables (elevation, temperature, precipitation) using diversity and specificity indices as well as network statistics, and (3) to identify certain myco-/photobiont associations that are representative for climatic conditions and therefore may qualify as bioindicators.

## Materials and methods

### Study area and investigated lichen specimens

Altogether 306 lecideoid lichen samples were collected along a latitudinal gradient (78–85.5°S) of the southwest Ross Sea coast (Antarctica, Fig. 1a) on siliceous rock in microhabitats providing the necessary life-enhancing features (Fig. 1e,f). Since no standardized climate data from local climate stations were available, climate data for the respective macroclimate of the study areas were used from the CHELSA database (Climatologies at high resolution for the earth’s land surface areas^49^). The sample sites were divided in five different main areas (Fig. 1b). Area 4 (Darwin Area) was then subdivided in subareas 4a and 4b, considering the wide range of climate conditions within this region (Fig. 1c). Site descriptions of the six regions are given in Table 1, geographical descriptions can be found at the Supplementary Table S1 online.

**Table 1:** Site descriptions of the six regions defined in the present study, including range of the coordinates of the sampling sites and areas, the number of sampling sites and the BIOCLIM variables BIO10 (mean temp. of the warmest quarter) and BIO12 (annual precipitation) per area.

	Sampling area	Range of coordinates of sampling sites	Number of sampling sites	Elevation mean (m.a.s.l.)	BIO10: mean temperature, warmest quarter (°C)	BIO12: precipitation, annual mean (mm)
Area 1	Scott Glacier/ Durham Point	S 85.54° W 151.15°	1	370.00	− 7.30	190.00
Area 2	Massam Glacier/Garden Spur	S 84.54°–84.56° W 174.91°–175.01°	2	182.92	− 6.96	113.00
Area 3	Mt. Kyffin, The Gateway, Mt. Harcourt	S 83.49°–83.83° E 170.79°–172.76°	6	774.72	− 8.20	104.37
Area 4a	Darwin Area: Diamond Hills, Brown Hills	S 79.84°–79.88° E 159.22°–159.39°	30	484.19	− 8.23	91.79
Area 4b	Darwin Area: Bartrum Basin, Smith Valley, Lake Wellman	S 79.75°–79.95° E 156.70°–158.67°	31	726.39	− 10.18	69.13
Area 5	McMurdo Dry Valleys	S 78.02°–78.17° E 163.62°–164.10°	102	589.42	− 6.24	145.04

The sampling strategy strongly depended on accessibility to the areas and length of the respective collection campaigns. All lichen specimens were collected in the field by using a fine chisel. The dry samples were stored close to the sites and afterward in the labs in a fridge at − 20 °C. In any case, all three collectors did a representative sampling of the available species in the remote areas.

147 samples of the genera *Carbonea, Lecanora, Lecidella* and *Lecidea* were collected at 70 different localities from the following sampling areas: Area 1, Scott Glacier/Durham Point; Area 2, Massam Glacier/Garden Spur; Area 3, Mt. Kyffin, Mt. Harcourt, The Gateway; Area 4a, Darwin Area (Diamond Hills, Brown Hills); Area 4b, Darwin Area (Bartrum Basin, Smith Valley and Lake Wellman) (Fig. 1a–c, Supplementary Table S1 online). To get a better coverage of the latitudinal gradient (Fig. 1a), additionally, 159 lichen samples (collected at 102 different localities) from the Area 5, McMurdo Dry Valleys (MDV), were obtained from the studies of Wagner et al.^10^ and Perez-Ortega et al.^50^, including the solely lecideoid lichen species of the genera *Carbonea, Lecanora, Lecidella, Lecidea* and *Rhizoplaca*. All newly processed specimen were determined by Ulrike Ruprecht. The entire lists of samples (including information on voucher-IDs, area, GPS data, species names of mycobionts, OTU-IDs of photobionts and accession numbers of the sequences) can be found at the Supplementary Tables S2–S4 online.

All voucher specimens are stored in the herbarium of the University of Salzburg (SZU) except for samples collected by Leopoldo G. Sancho which are deposited in the MAF herbarium of the Botany Unit, Fac. Farmacia, in Madrid.

The study complied with the institutional and the national guidelines. The collecting events were conducted in accordance with the McMurdo Dry Valley Antarctic Specially Managed Area manual (New Zealand Ministry of Foreign Affairs and Trade). The PIs of the projects had current permits to both collect and import lichens into NZ, granted by the Ministry of Foreign Affairs and Trade (MFAT) and by The Ministry of Primary Industries (MPI). All of these were in place during the collection of these samples.

### DNA-amplification, primer-design, sequencing and phylogenetic analyses

Total DNA was extracted from individual thalli by using the DNeasy Plant Mini Kit (Qiagen) following the manufacturer’s instructions. For all samples, the internal transcribed spacer (ITS) region of the mycobionts’ and photobionts’ nuclear ribosomal DNA (nrITS) were sequenced and amplified. Also, additional markers were amplified: for the mycobionts the mitochondrial small subunit (mtSSU) and the low-copy protein coding marker *RPB1*; for the photobionts, the chloroplast-encoded intergenic spacer (psbJ-L) and part of the cytochrome oxidase subunit 2 gene (COX2). This was done using specific primers and PCR-protocols in our project framework^47^ (Supplementary Table S5 online). Unpurified PCR-products were sent to Eurofins Genomics/Germany for sequencing.

Phylogenetic analyses were performed according to Muggia et al.^51^, Ruprecht et al.^48^ and Wagner et al.^10^ for both symbionts: in each case a single marker (nrITS) and a three marker phylogeny (mycobionts: nrITS/mtSSU/*RPB1*; photobiont: nrITS/psbJ-L/COX2) (Supplementary Table S6 online). In order to be able to use also samples with an incomplete marker set, both the multi-marker phylogeny (with a reduced number of samples) as well as the ITS marker phylogeny (complete data set) were calculated and compared subsequently, for each symbiont respectively.

### Analysis of spatial distribution

Unless stated otherwise, analysis was conducted in R^52^ (version 3.6.3, https://www.r-project.org); figures where produced using the R package ggplot2^53^ and processed using Adobe Photoshop (version 22.2.0., https://www.adobe.com).

Based on data from CHELSA (Climatologies at high resolution for the earth’s land surface areas^49^), the 19 BIOCLIM variables^54^ were calculated for each sample point using the R functions raster() and extract() of the package raster^55^. These variables are derived variables from the monthly minimum, maximum, mean temperature and mean precipitation values, developed for species distribution modeling and related ecological applications^49^. For the analyses of this study, BIO10 (mean temperature of the warmest quarter) and BIO12 (annual precipitation) were chosen, as these two variables showed the strongest correlations with the diversity and specificity indices (see below).

For analyzing the spatial distribution of the lichen samples, alpha, beta and gamma diversity values were calculated. Alpha diversity reflects the diversity of individuals *in* local assemblages whereas beta diversity reflects the diversity *of* the local assemblages. Gamma diversity gives the overall diversity of all individuals found within a given area^56^. These diversity indices were calculated separately for mycobiont species and photobiont OTUs, using the R functions AlphaDiversity(), BetaDiversity() and GammaDiversity() of the package entropart^56^ which give reduced-bias diversity values. Estimators of diversity may be biased because of unseen species and also because they are not linear functions of probabilities^57^. Concerning the order of diversity, we chose *q* = 1 for Shannon diversity, which considers both species richness as well as evenness; for weights *w*_*i*_ = *n*_*i*_*/n* with *n*_*i*_, number of samples in area *i* and *n*, the total number of samples was used. Next, alpha diversity was analyzed for correlations with the following variables: elevation, latitude, BIO10 and BIO12. Additionally, for both mycobiont species and photobiont OTUs, sample coverage was calculated, representing a useful indicator of sampling quality e.g.,^58^: it gives the probability for a species of the community to be observed in the actual sample. This was done by applying the base function summary() on a MetaCommunity object (R package entropart)^56^. Furthermore, based on sample coverage, rarefaction/extrapolation curves were constructed, using the R function iNEXT of the same package^59,60^. Using the R function estimateD() alpha diversity values with a specified level of sample coverage of 95% were calculated, in order to make comparisons between the different areas.

To determine whether mycobiont species or photobiont OTU community composition are related to environmental variables (elevation, BIO10 and BIO12), a constrained analyses of principal coordinates were conducted, using the R function capscale() of the package vegan^61^ (distance: Bray Curtis). Prior to analysis, to standardize species composition data (convert species abundances from absolute to relative values), a Hellinger transformation was performed on the community matrix, using the R function decostand() of the package vegan^61^. The variance explained by constrained ordination was tested by a Monte Carlo permutation test, using the R function anova() of the package vegan^61^.

A Mantel test was performed to test whether the differences in mycobiont species and photobiont OTU community composition between samples are related to physical distance, using the R function mantel() of the package vegan^61^.

### Haplotype analysis

In order to ensure that the entire data set could be processed, all further analyses were carried out using only complete sequences of the marker ITS for all calculations. The number of haplotypes, *h,* of the different mycobiont species and photobiont OTUs was determined using the function haplotype() of the R package pegas^62^. Haplotype networks were computed, using the function haploNet() of the R package pegas^62^ for mycobiont species and photobiont OTUs with *h* ≥ 2 and at least one haplotype with *n* ≥ 3 (*Carbonea* sp. 2, *Lecanora fuscobrunnea*, *Lecidea cancriformis, Lecidella greenii, Lecidella siplei, Lecidella* sp. nov2 and *Rhizoplaca macleanii,* as well as *Tr*_A02, *Tr*_I01 and *Tr*_S02). The frequencies were clustered in 10% ranges, for example the circles of all haplotypes making up between 20 and 30% have the same size. Additionally, for the most common mycobiont species *L. cancriformis* and the photobiont OTU *Tr*_S02, haplotype networks based on multimarker data sets were calculated, to show that the distribution of haplotypes remains congruent.

### Diversity and specificity indices of mycobiont species and photobiont OTUs

The haplotype as well as the nucleotide diversity were calculated for each identified mycobiont species and photobiont OTU with more than one sample, using the functions hap.div() and nuc.div() of the R package pegas^62^, respectively. The haplotype diversity, *Hd,* represents the probability that two randomly chosen haplotypes are different^63^, the nucleotide diversity, π*,* gives the average number of nucleotide differences per site between two randomly chosen DNA sequences^64^. Additionally, the ratio of the number of haplotypes *h* divided by the number of samples *N* was calculated.

Furthermore, different metrics for quantifying the phylogenetic species diversity and the specificity of the mycobiont species and photobiont OTUs towards their interaction partners were calculated. Those included the indices *NRI* (net relatedness index), *PSR* (phylogenetic species richness) and the Pielou evenness index *J*′. *(Note: to make interpretation similar to the other metrics, for further analyses* 1 – *J*′ *instead of* J’ *was used.)* An overview of these diversity metrics is given in Supplementary Table S7 online.

In order to analyze the correlation of these diversity metrics with environment, for every mycobiont species and photobiont OTU with *n* ≥ 10 (*Carbonea* sp. 2, *C. vorticosa, Lecanora fuscobrunnea, Lecidea cancriformis, L. polypycnidophora, Lecidella greenii, L. siplei, Rhizoplaca macleanii* and *Tr*_A02, *Tr*_I01, *Tr*_S02, *Tr*_S15, *Tr*_S18) the mean values of the sample locations of the following variables were calculated: elevation, latitude, BIO10 and BIO12.

### Analysis of mycobiont–photobiont associations

To analyze the associations between mycobiont species and photobiont OTUs, bipartite networks were computed, using the R function plotweb() of the package bipartite^65^ This was done for each area separately. Additionally, for each bipartite network, the index *H*_2_′ was calculated. *H*_2_′ is derived from Shannon entropy and characterizes the degree of complementary specialization of partitioning among the two parties of the network. It ranges from 0 for the most generalized to 1 for the most specialized case und was computed using the R functions H2fun() of the package bipartite^65^.

Usually, in the context of bipartite networks, also the *d*′ value (specialization index) is computed. This value was originally defined for pollination networks and calculates how strongly a species deviates from a random sampling of interacting partners available^66^. Thus, in the case of lichens, the *d*′ value of a mycobiont species is based on the assumption that for every site of a sampling area, the whole set of photobiont OTUs basically is available. As this is not true for this study, this index was not included.

## Results

### Phylogenetic analysis

For both the mycobiont and photobiont molecular phylogenies from multi-locus sequence data (nrITS, mtSSU and *RPB1* for the mycobiont (140 samples) and nrITS, psbJ-L and COX2 for the photobiont (139 samples) were inferred (Supplementary Figs. S1 and S3 online). Additionally, phylogenies based solely on the marker nrITS were calculated (Supplementary Figs. S2 and S4 online), to include samples where the additional markers were not available. Both analyses include only accessions from the study sites (Fig. 1; Table 1). The phylogenies based on the multi-locus data were congruent to the clades of the phylogenies based on the marker nrITS. Thus, in the following, the focus will be only on the latter.

#### Mycobiont

The final data matrix for the phylogeny based on the marker nrITS comprised 306 single sequences with a length of 550 bp. It included sequences of the families Lecanoraceae and Lecideaceae. The phylogenetic tree was midpoint rooted and shows a total of 19 strongly supported clades on species level, assigned to five genera. The backbone is not supported and therefore the topology will not be discussed. All genera are clearly assigned to their family level and are strongly supported. Only *Lecanora physciella* forms an extra clade as sister to the families Lecideaceae and Lecanoraeae, which is not the case at the multimarker phylogeny. *L. physciella* has still an uncertain status, because of morphological similarities to both sister families^6^. The clade of the genus *Lecidea* revealed seven species (*L. andersonii*, *L. polypycnidophora*, *L.* UCR1, *L.* sp. 5, *L. lapicida*, *L. cancriformis* and *L.* sp. 6), *Lecanora* five species (*L. physciella*, *L.* sp. 2, *L. fuscobrunnea*, *L.* cf. *mons*-*nivis*, *L.* sp. 3), *Carbonea* three species (*C.* sp. URm1, *C. vorticosa*, *C.* sp. 2), and *Lecidella* three species (*L. greenii*, *L. siplei*, *L.* sp. nov2). The samples allocated to the genus *Rhizoplaca* were monospecific (*R. macleanii*). The taxonomical assignment of the obtained sequences were based on the studies of Ruprecht et al.^48^ and Wagner et al.^10^.

#### Photobiont

The final data matrix for the phylogeny based on the marker nrITS comprised 281 single sequences with a length of 584 bp. The phylogenetic tree was midpoint rooted and shows six strongly supported clades, assigned to seven different OTU levels^67^, using the concept of Muggia et al.^51^ and Ruprecht et al.^48^. All of the OTUs belong to the genus *Trebouxia* (clades A, I, S), comprising *Tr_*A02, *Tr*_A04a, *Tr*_I01, *Tr*_I17, *Tr_*S02, *Tr_*S15 and *Tr_*S18. Photobiont sequences taken from Perez-Ortega et al.^50^, which were labelled only with numbers, were renamed to assign them to the appropriate OTUs^48^.

### Analysis of spatial distribution

In general, the most common mycobionts species were *Lecidea cancriformis* (94 of the 306 samples), *Rhizoplaca macleanii* (51 samples) and *Lecidella greenii* (37 samples), followed by *Carbonea* sp. 2 (13 samples), *C. vorticosa* (11 samples), *Lecidea polypycnidophora* (10 samples) and *Lecidella siplei* (10 samples; see Supplementary Fig. S5 online). Nine mycobiont species were found exclusively in area 5 (MDV, 78°S): *Carbonea vorticosa*, *Lecanora* cf. *mons-nivis*, *L.* sp. 2, *Lecidea lapicida*, *L. polypycnidophora*, *L*. sp. 5, *L*. sp. 6*, L*. UCR1 and *Rhizoplaca macleanii.* On the other hand, only *Lecidea cancriformis* was found in all the six areas; *Lecanora fuscobrunnea* was present in all the areas with the exception of area 2.

The most common photobiont OTUs were *Tr*_A02 (165 of the 281 samples) and *Tr*_S02 (59 samples), both of them occurring in all the six different areas, followed by *Tr*_S18 (32 samples), *Tr*_S15 (10 samples, confined to area 5) and *Tr*_I01 (10 samples). However, of the 149 photobiont accessions of area 5, 134 (89.93%) were assigned to *Tr*_A02. This percentage is much higher than in the other areas (area 1: 44.44%, area 2: 69.23%, area 3: 21.74%, area 4a: 7.69%, area 4b: 6.67%), even if those samples with mycobionts occurring exclusively in area 5 (see above) were excluded (76.56% of the 64 remaining samples are assigned to *Tr*_A02).

The alpha, beta and gamma diversity values are given in Table 2. For the mycobionts, the alpha diversity of the communities was the highest in area 5 (8.93, which results in nine species) and the lowest in area 4b (two species, 1.88). In contrast, for the photobionts, the lowest alpha diversity value was found in area 5 (two OTUs, 1.50) and the highest in area 4a (four OTUs, 4.06). Thus, referring to this, area 5 plays a remarkable role: compared to the other areas, it shows the highest diversity of mycobiont species on the one hand and the lowest diversity of photobiont OTUs on the other hand.

**Table 2:** Number of lichen samples, number of identified mycobiont species and photobiont OTUs, as well as alpha, beta and gamma diversity values of mycobiont species/photobiont OTUs for the different areas.

Area	Number of lichen samples	Mycobiont species				Photobiont OTUs			
		Number of identified species	Alpha diversity	Beta diversity	Gamma diversity	Number of identified OTUs	Alpha diversity	Beta diversity	Gamma diversity
1	28	7	5.23	1.69	9.92	3	2.28	1.64	3.35
2	13	5	5.48			3	2.20		
3	27	7	5.99			5	3.70		
4a	48	6	3.55			6	4.06		
4b	31	2	1.88			4	2.36		
5	159	16	8.93			4	1.50		

The beta diversity values (diversity of local assemblages) for mycobiont species and photobiont OTUs are quite similar (1.69 and 1.64, respectively). This is in contrast to gamma diversity values: the overall diversity for the different areas within the whole region is much higher for the mycobionts (ten species, 9.92) than for the photobionts (three OTUs, 3.35).

For mycobionts, the overall sample coverage equals to 0.993. That means that the probability for an individual of the community to belong to a sampled species is 99.3%, or, from another point of view, the probability for an individual of the whole community to belong to a species that has not been sampled is 0.7%. The sample coverage is highest for area 4b (1.000) and lowest for area 2 (0.771). Sample coverage values of the other areas are in between (area 1: 0.895, area 3: 0.931, area 4a: 0.939, area 5: 0.981). The rarefaction/extrapolation curves for the mycobiont species (see Supplementary Fig. S6a) suggest that for any sample size up to the specified level of sample coverage of 0.95, alpha diversity within area 4b is significantly lower than alpha diversity within any other area, and alpha diversity within area 5 is significantly greater than that of area 4a and 4b (based on 95% confidence intervals).

For photobionts, the overall sample coverage as well as the sample coverages of area 1, area 2, area 3, area 4b as well as area 5 is equal 1.000. Only the sample coverage of area 4a (0.951) differs. The rarefaction/extrapolation curves for the photobiont OTUs (see Supplementary Fig. S6b) suggest that for any sample size up to the specified level of sample coverage of 0.95, alpha diversity within area 1 is significantly lower than alpha diversity of area 3 and 4a and significantly greater than that of area 5. Alpha diversity of area 5 is significantly lower than that of area 1, area 3 and area 4a.

### Influence of environmental factors (elevation, precipitation and temperature)

First, the proportion of the OTU *Tr*_A02 samples was significantly correlated to BIO10 means of the areas (R = 0.87, *p* = 0.022; see Supplementary Fig. S7 online): the higher the temperature mean values of the warmest quarter of an area, the higher the proportion of samples containing photobionts that are assigned to *Tr*_A02.

The alpha diversity values of mycobiont species significantly positively correlated with BIO10 (R = 0.88, *p* = 0.021; see Supplementary Fig. S8 online): the higher the temperature mean values of the warmest quarter, the higher the mycobiont diversity within this particular area.

Furthermore, the differences in mycobiont species community composition were significantly related to BIO10 (constrained principal coordinate analysis: F = 14.7137, *p* = 0.001, see Supplementary Fig. S9 online), BIO12 (F = 2.7535, *p* = 0.012), elevation (F = 2.5108, *p* = 0.025) and the geographic separation of the samples (Mantel statistic r = 0.1288, *p* = 0.0002).

The differences in community composition of photobiont OTUs were related significantly to BIO10 (constrained principal coordinate analysis: F = 48.5952, *p* = 0.001, see Supplementary Fig. S10 online), BIO12 (F = 4.4848, *p* = 0.008), elevation (F = 6.8608, *p* = 0.002), and physical distance (Mantel statistic r = 0.4472, *p* = 0.0001).

### Haplotype analysis

**Figure 2:**
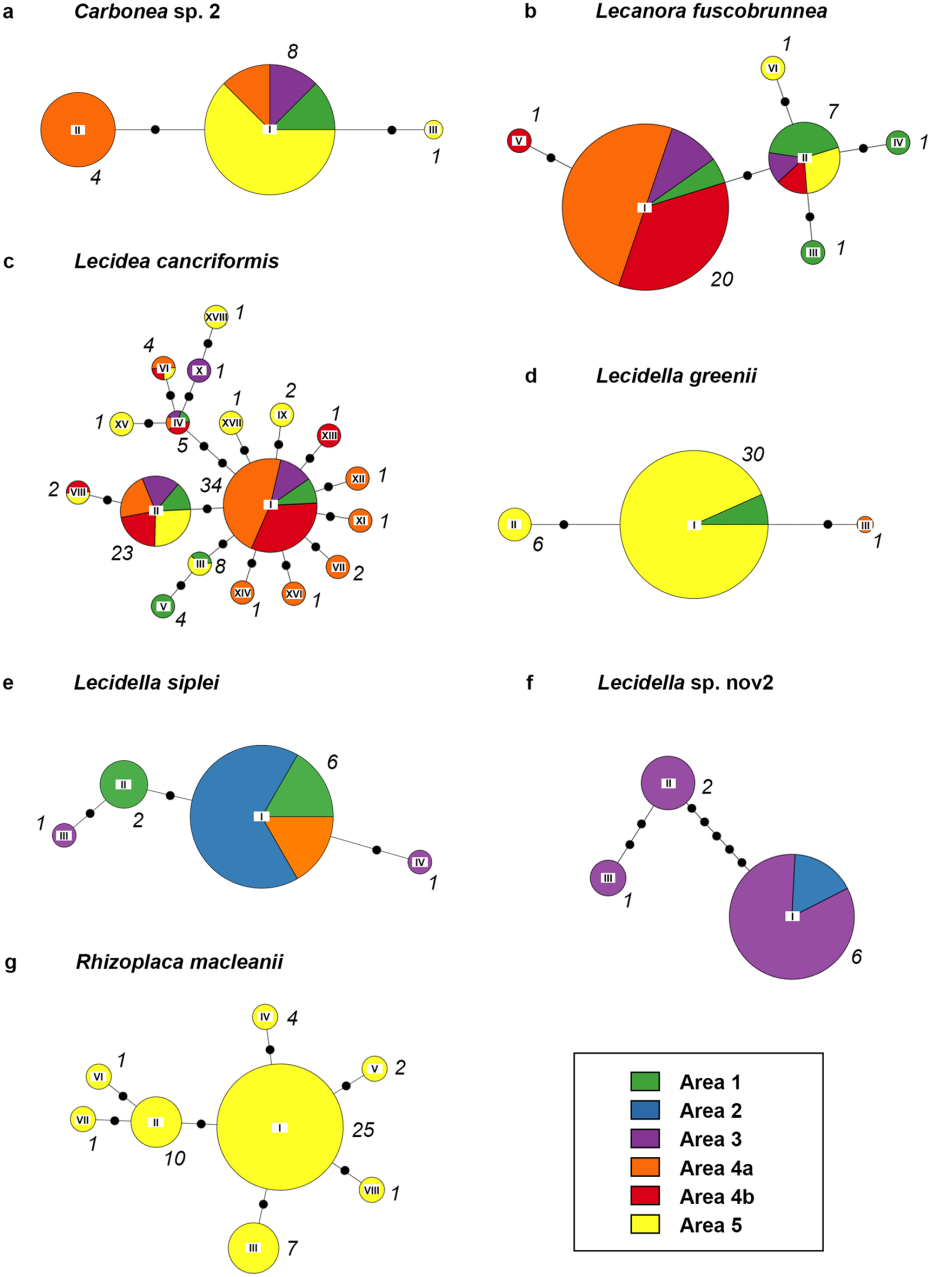
Haplotype networks of mycobiont species with h ≥ 2 and at least one haplotype with n ≥ 3, showing the spatial distribution within the different areas, based on nrITS data. (**a**) *Carbonea* sp. 2, (**b**) *Lecanora fuscobrunnea*, (**c**) *Lecidea cancriformis*, (**d**) *Lecidella greenii*, (**e**) *Lecidella siplei*, (**f**) *Lecidella* sp. nov2, (**g**) *Rhizoplaca macleanii*. Roman numerals at the center of the pie charts refer to the haplotype IDs; the italic numbers next to the pie charts give the total number of samples per haplotype. The circle sizes reflect relative frequency within the species; the frequencies were clustered in ten (e.g. the circles of all haplotypes making up between 20 and 30% have the same size). Note: only complete sequences were included.

**Figure 3:**
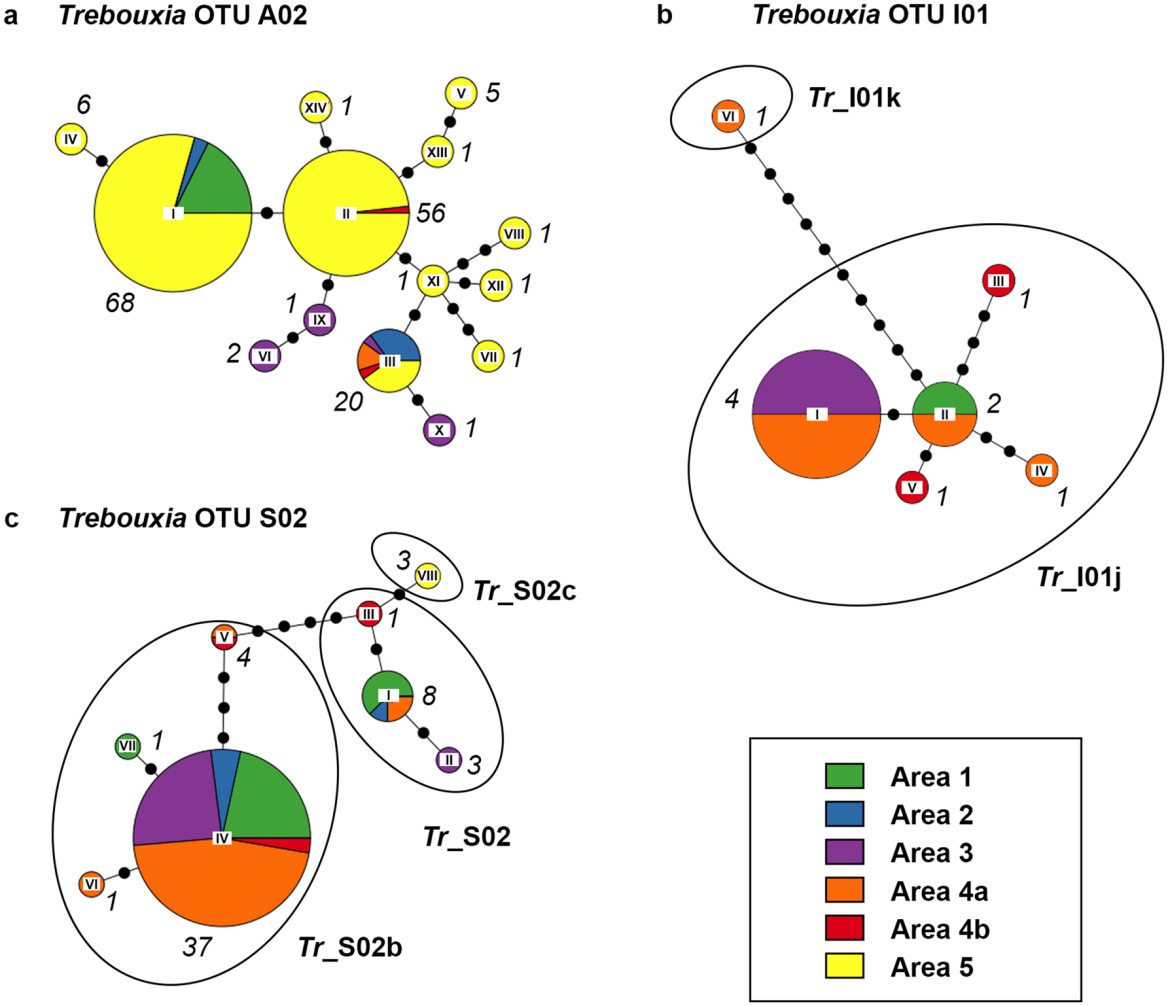
Haplotype networks of photobiont OTUs with h ≥ 2 and at least one haplotype with n ≥ 3, showing the spatial distribution within the different areas, based on nrITS data. (**a**) *Tr*_A02, (**b**) *Tr*_I01, (**c**) *Tr*_S02. Roman numerals at the center of the pie charts refer to the haplotype IDs; the italic numbers next to the pie charts give the total number of samples per haplotype. The circle sizes reflect relative frequency within the species; the frequencies were clustered in ten (e.g. the circles of all haplotypes making up between 20 and 30% have the same size). Note: only complete sequences were included.

Haplotype networks were computed for the mycobiont species and photobiont OTUs with *h* ≥ 2 and at least one haplotype with *n* ≥ 3 (*Carbonea* sp. 2, *Lecanora fuscobrunnea*, *Lecidea cancriformis, Lecidella greenii, L. siplei, L.* sp. nov2 and *Rhizoplaca macleanii,* as well as *Tr*_A02, *Tr*_I01 and *Tr*_S02), in both cases based on nrITS sequence data (Figs. 2, 3). The samples of *Carbonea vorticosa* (11) were all assigned to a single haplotype, which was also true for *Lecidea polypycnidophora* (10 samples), *Tr*_S15 (10 samples) and *Tr*_S18 (32 samples). Figure 3b, c illustrate the subdivision of *Tr*_I01^51^ into *Tr*_I01j^35,48^ and *Tr*_I01k (in this study), and the subdivision of *Tr*_S02 into *Tr*_S02^35^, and *Tr*_S02b and *Tr*_S02c^48^.

The haplotype networks include pie charts showing the occurrence of the different haplotypes within the different areas. All haplotypes of *Rhizoplaca macleanii* are restricted to area 5, as well as *Lecidella greenii* mainly to area 5 and areas 1 and 4a, and *Lecidella* sp. 2 to areas 2 and 3. However, all other species do not suggest a spatial pattern with different haplotypes being specific for different areas. Moreover, the distribution turned out to be rather unspecific, with a great part of the haplotypes found in multiple areas. For the sake of completeness, additionally, haplotype networks based on multi-locus sequence data were computed for the most abundant mycobiont species and photobiont OTU with multi-locus data available (*Lecidea cancriformis* and *Tr*_S02). Not surprisingly, those networks show a greater number of different haplotypes, but they also do not allow conclusions concerning spatial patterns of area specific haplotypes (see Supplementary Fig. S11 online).

### Diversity and specificity indices of mycobiont species and photobiont OTUs

The diversity and specificity indices for the different mycobiont species and photobiont OTUs are given in Supplementary Table S8 online.

**Figure 4:**
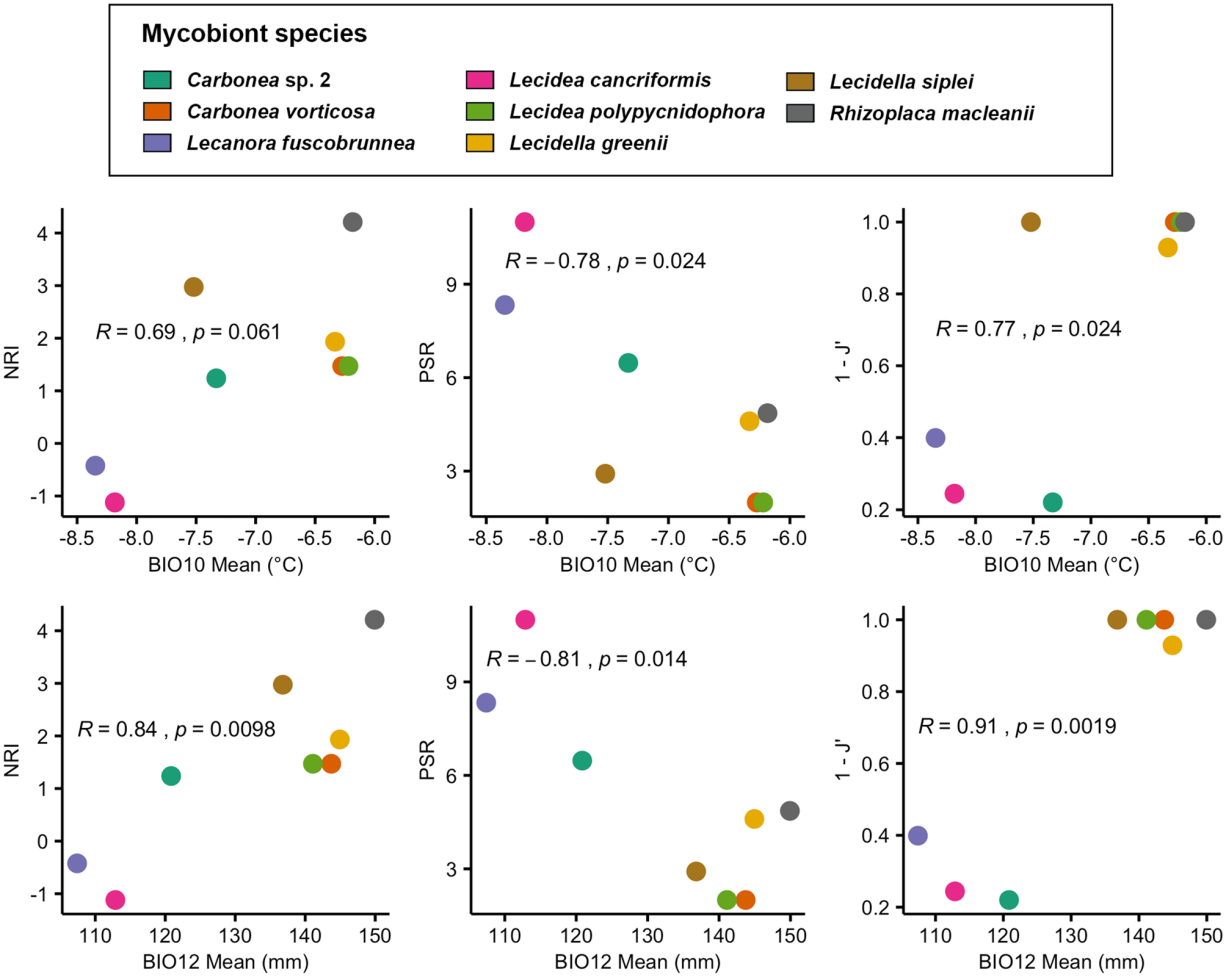
Correlation plots. Specificity indices *NRI* (net relatedness index), *PSR* (phylogenetic species richness and 1 – *J*′ (Pielou evenness index) against mean values of BIO10 (mean temperature of warmest quarter) and BIO12 (annual precipitation) for mycobiont species with n ≥ 10.

For the sample locations of mycobiont species with *n* ≥ 10, BIO10 was strongly correlated to the specificity indices *NRI* (net relatedness index) and significantly correlated to *PSR* (phylogenetic species richness) and *1 – J*′ (Pielou evenness index). BIO12 was significantly correlated to *NRI*, *PSR* and *1 – J*′. Figure 4 illustrates these correlations: the higher the BIO10 and BIO12 mean values, the higher was the *NRI* (phylogenetic clustering of the photobiont symbiotic partners), the lower was the *PSR* (increased phylogenetically relatedness of photobiont symbiotic partners) and the higher was *1 – J*′ (less numerically evenness of the photobiont symbiotic partners). Thus, for the mean values of the sample locations of a mycobiont species, a comparatively high temperature of the warmest quarter and high annual precipitation occurs with associated photobionts that are phylogenetically clustered and closer related to each other. The lowest values of *NRI* and the highest values of *PSR* were developed by *Lecidea cancriformis* and *Lecanora fuscobrunnea*, which also showed the lowest BIO10 and BIO12 mean values at their sample sites. On the contrary, the highest values of *NRI* and *PSR* were developed by *Rhizoplaca macleanii*, which also had the highest BIO10 and BIO12 means.

**Figure 5:**
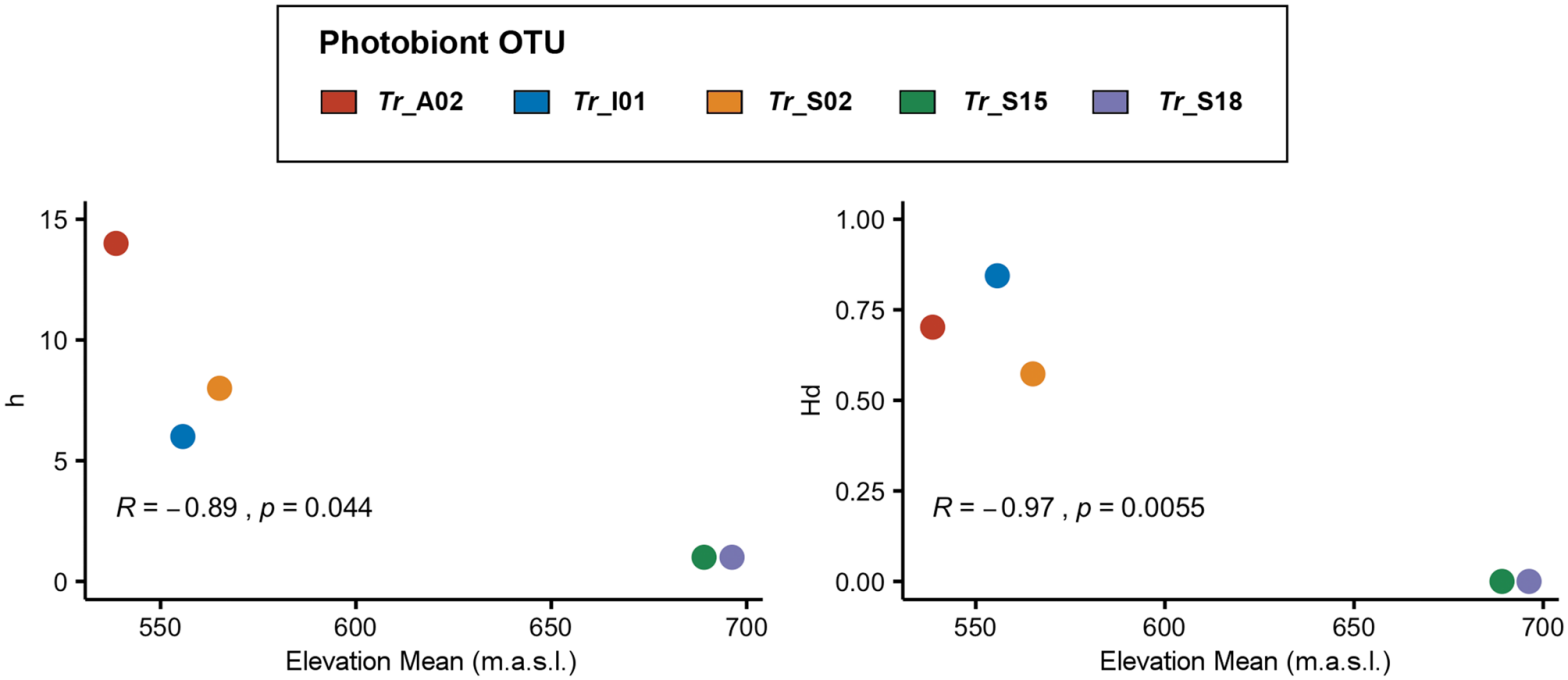
Correlation plots. Diversity indices *h* (number of haplotypes) and *Hd* (haplotype diversity) against mean elevation of sample sites for photobiont OTUs with n ≥ 10.

For the sample locations of photobiont OTUs with *n* ≥ 10, elevation significantly negatively correlated with *h* (number of haplotypes) and *Hd* (haplotype diversity): the higher the mean elevation of sample sites, the lower the number of haplotypes and the lower the probability that two randomly chosen haplotypes are different (Fig. 5). The highest values of h and *Hd* were shown by *Tr*_A02, *Tr*_I01 and *Tr*_S02, which occurred at sample sites with comparatively low elevations. In contrast, *Tr*_S15 and *Tr*_S18 occurred at very high elevations and showed very low values of *h* and *Hd*.

### Analysis of mycobiont–photobiont associations

**Figure 6:**
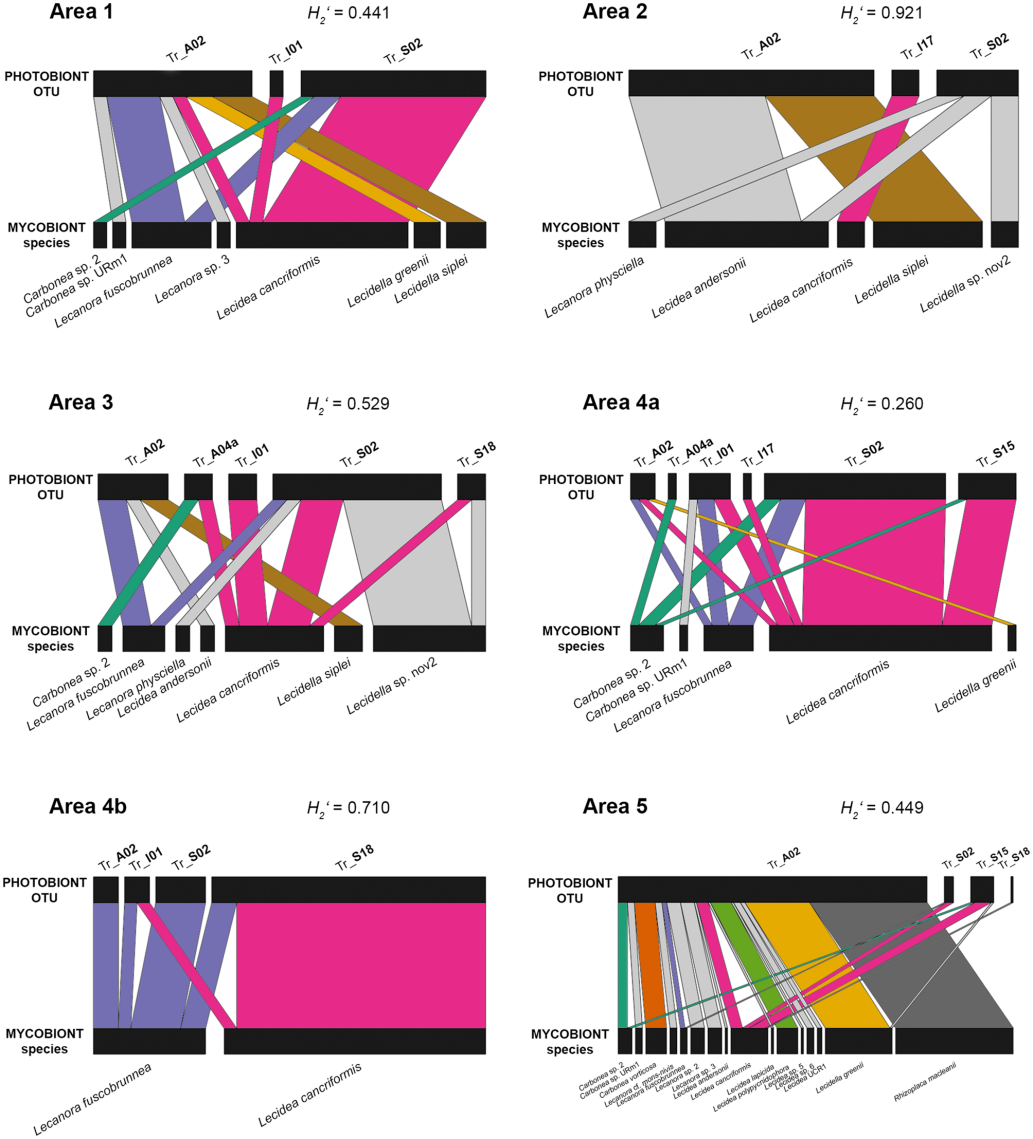
Bipartite networks showing the associations between mycobiont species and photobiont OTUs for the different areas. Rectangles represent species/OTUs, and the width is proportional to the number of samples. Associated species/OTUs are linked by lines whose width is proportional to the number of associations.

Bipartite networks were calculated for all associations between mycobiont species (lower level) and the respective photobiont OTUs (higher level) for all areas (Fig. 6). The *H*_*2*_′ value (overall level of complementary specialization of all interacting species) was highest in area 2 (0.921), indicating a network with mostly specialized interactions: within this network, with the exception of *Lecidea andersonii*, the mycobiont species are associated exclusively with one single photobiont OTU. The second highest *H*_*2*_′ value was developed by area 4b (0.710); in contrast, area 4a showed the lowest *H*_*2*_′ value (0.260), with the most abundant mycobiont species *Lecidea cancriformis* showing associations with five different photobiont OTUs. The *H*_*2*_′ values of area 1, area 3 and area 5 indicate medium specification.

In addition, the bipartite networks illustrate the different occurrence of mycobiont species and photobiont OTUs within the different areas: For example, in area 1 (and area 2), five (seven) different mycobiont species are associated with only three different photobiont OTUs. In contrast, in area 4b, only two different mycobiont species are associated with four different photobiont OTUs. In area 5, the number of associated photobiont OTUs is also four, but those four OTUs are associated with 16 different mycobiont species.

The network matrix giving all the associations between the mycobiont species and photobiont OTUs is presented in Supplementary Table S9 online.

## Discussion

The present study investigated the diversity of lecideoid lichens at six different sample areas along a latitudinal gradient (78°S–85.5°S) along the Transantarctic Mountains, Ross Sea region, in continental Antarctica. The distribution of the different mycobiont species and photobiont OTUs differed considerably between the six sample areas, which is expressed in alpha diversity values (Supplementary Fig. S8 online). The extreme climate (lowest mean temperatures of the warmest quarter, lowest precipitation) at the areas 4a & b (Darwin Area, ~ 79.8°S) was reflected in the lowest alpha diversity of mycobionts, and comparatively high alpha diversity of photobiont OTUs. On the other hand, in the climatically mildest area 5 (McMurdo Dry Valleys, 78°S, highest mean temperature, second highest precipitation), the highest alpha diversity of mycobionts and the lowest alpha diversity of photobiont OTUs was found. The rarefaction/extrapolation curves based on sample coverages confirmed the significance of these differences of the alpha diversity within area 5 and area 4a & b (mycobiont), and within area 5 and area 4a (photobiont), respectively. The number of different photobiont OTUs identified per area is comparatively at a similar level (varying between three and six different groups, Table 2), which is remarkable when considering the great differences in sample sizes (varying between 13 samples (area 2) and 159 samples (area 5), Table 2).

These results are partially consistent with the findings of Colesie et al.^8^, who previously reported that macroclimatic conditions along the latitudinal gradient are not linear as well as the species distribution of terrestrial vegetation, and concluded that this irregularity is most likely caused by the shape of the landscape and the water availability (melting water) of the microhabitats^13^. The area at Diamond Hill (79.9°S, part of area 4a, Darwin area, Fig. 1c) addressed in Colesie et al.^8^ showed the lowest species diversity along the latitudinal gradient, which was at least confirmed for area 4b in the current study. Areas 4a and especially 4b are characterized by the harshest climatic conditions such as very low humidity and temperatures, and species numbers in relation to the number of samples are also lowest there; the two generalist species *Lecidea cancriformis* and *Lecanora fuscobrunnea* are dominant. Milder areas, further south (83.5–85.5°S), and the McMurdo Dry Valleys (78°S), enable a higher species diversity^13,50^. The higher diversity at the southern sites, particularly Mt. Kyffin, appears in part to be due to the occurrence of relic species^13^. However, Colesie et al.^8^ suggested that physical barriers could be the reason for the low diversity in the Darwin area, but the unspecified haplotype distribution of the widespread species suggest that this is not the case. Since the substrate at all sites is siliceous and there are no other obvious limiting factors, the most likely reason for the limited occurrence of certain species is primarily dependent on abiotic factors, in particular, the environmental conditions caused by geography and macroclimate^33^.

The uniformity of photobiont OTUs in area 5 is mainly due to a strong dominance (89.93%) of the OTU *Tr_*A02 which occurred in all the six sampling areas. The proportion of photobiont accessions assigned to *Tr*_A02 was significantly correlated to the mean value of BIO10 (mean temperature of the warmest month) of the areas. Thus, higher temperatures are related to a higher relative abundance of *Tr*_A02, and colder temperatures to a higher relative abundance of other photobiont OTUs. This result is in basic agreement with the previous study of Wagner et al.^10^. The community composition of both, mycobiont species as well as photobiont OTUs, is significantly related to elevation, BIO10 and BIO12 (annual precipitation). Thus, as sampling sites become more dissimilar in terms of elevation, BIO10 or BIO12, they also become more dissimilar in terms of community composition. These findings are partially supported by Rolshausen et al.^40^ who surveyed *Trebouxia* communities in temperate climates, suggesting that photobiont OTUs are replaced by others as environmental conditions change. In addition, significant correlations emerged between the composition of mycobiont and photobiont communities and the geographic separation of the samples: The further the sampling sites are spatially separated, the more dissimilar the corresponding communities become, which is in agreement with Fernandez-Mendoza et al.^68^.

Furthermore, the specificity of mycobiont species towards their photobiont partners was shown to be related to environmental variables; these findings are partially in agreement with the studies of Peksa and Skaloud^36^, Singh et al.^22^ and Rolshausen et al.^34^, who reported climate as well as substrate as a selective pressure in terms of increased specificity of mycobiont-photobiont interactions. However, the current study has shown that a higher value of BIO10 correlated with a higher phylogenetic clustering of the symbiotic partners of a single mycobiont species (higher 1 – *J*′ and *NRI* values) and a closer phylogenetic relatedness of these photobionts (lower *PSR* values). Similarly, the specificity of mycobiont species towards their photobiont symbiotic partners also correlated with BIO12 mean values: A higher value of BIO12 is related to higher values of 1 – *J*′ and *NRI* and to lower values of *PSR*. Consequently, the mycobiont species with *n* ≥ 10 showing the highest BIO10 and BIO12 mean values at its sample locations (*Rhizoplaca macleanii*) also had the highest value of *NRI* and a rather low value of *PSR*, as it was solely associated with *Tr*_A02. On the other hand, the two mycobiont species with *n* ≥ 10 showing the lowest mean values of BIO10 and BIO12 (*Lecanora fuscobrunnea* and *Lecidea cancriformis)* exhibited the lowest values of *NRI* and the highest values of *PSR*, as they had associations with the phylogenetically distinct photobiont OTUs *Tr_*A02, *Tr_*I01, *Tr_*S02 and *Tr_*S18 (*Lecanora fuscobrunnea*) or all seven *Trebouxia* OTUs of this study (*Lecidea cancriformis*), respectively. Additionally, *Lecanora fuscobrunnea* and *Lecidea cancriformis* were the two most widespread species that occurred in five of the six (*L. fuscobrunnea*) or all the six different areas (*L. cancriformis*). This result is in agreement with former studies that had shown that *L. cancriformis* is able to associate with all known photobiont OTUs, and is one of the most widespread lichens in continental Antarctica^10,16,69,70^. Previous studies suggested that a higher photobiont diversity within a single lichen species is indicative of a lower selectivity by the mycobiont, and that this condition is related to enhanced colonization ability^71–73^. According to a model developed by Yahr et al.^74^, selectivity may vary between habitats and enable lichens to select a photobiont that is well adapted to conditions of the local environmental. These photobiont switches were suggested to increase the geographical range and ecological niche of lichen mycobionts, but may also lead to genetic isolation between mycobiont populations and thus drive their evolution^68^. More generally, flexibility concerning the partner choice has been considered to be an adaptive strategy to survive harsher environmental conditions^22,38,75,76^.

Dal Grande et al.^33^ reported elevational preferences for some *Trebouxia* taxa at the OTU level at a mountain range in central Spain, covering an elevational gradient of 1400 m. Additionally, in the present study, the mean elevation of photobiont OTUs were negatively correlated to differences in diversity indices: the dominant photobiont OTU *Tr*_A02, occurring in all the six different areas, exhibited the lowest mean value of elevation of sample sites and had the highest number of haplotypes (*h*) and the highest value of haplotype diversity (*Hd*). On the other hand, the OTUs *Tr*_S15 and *Tr*_S18 had the highest mean elevations and the lowest values of *h* and *Hd*. Thus, higher mean elevation of photobiont OTUs were significantly related to a lower number of haplotypes (*h*) and a lower haplotype diversity (*Hd*).

## Conclusions

Lichens and their myco-/photobiont associations clearly show environmental preferences and therefore are useful as bioindicators. The *Trebouxia* OTU A02 occurred in all the six different areas and was dominant in milder areas, whereas in colder areas, a higher relative abundance of other *Trebouxia* OTUs was found. Accordingly, mycobiont species occurring in milder areas (like *Carbonea vorticosa*, *Lecidella greenii* and *Rhizoplaca macleanii*) are almost exclusively associated with *Tr*_A02, while the generalist mycobiont species *Lecidea cancriformis* und *Lecanora fuscobrunnea*, occurring in a broad range of climatically different environments, show associations with phylogenetically distinct photobiont OTUs. However, if they are the only lecideoid lichen species present in certain areas, then they are also meaningful bioindicators of extreme climatic conditions.

## Supplementary Information

Supplementary Information 1.

Supplementary Information 2.
