# Supplementary material for: Macroclimatic conditions as main drivers for symbiotic association patterns in lecideoid lichens along the Transantarctic Mountains, Ross Sea region, Antarctica
Source: Sci Rep. 2021 Dec 6;11:23460. doi: 10.1038/s41598-021-02940-6 (PMC8648759; doi:10.1038/s41598-021-02940-6)
Supplement: Supplementary file 1 — Supplementary Information 1. [file 41598_2021_2940_MOESM1_ESM.pdf]

Supplementary Material 1: Tables

Contents

	Page
Supplementary Table S1	2
Supplementary Table S2	3
Supplementary Table S3	7
Supplementary Table S4	9
Supplementary Table S5	13
Supplementary Table S6	13
Supplementary Table S7	14
Supplementary Table S8	15
Supplementary Table S9	16
References	16

Supplementary Table S1. Geographical description of sampling sites.

	Sampling area	Range of coordinates of sampling sites	Collected by	Geographical description
**Area 1**	Scott Glacier/ Durham Point	S 85.54°<br>W 151.15°	Leo Sancho (2011)	**Durham Point** emerges as a big cliff closed to the confluence of Scott Glacier with the Ross Ice Shelf and it is surrounded by frozen lakes. The substrate is predominantly made up of crystalline plutonic (granite) or metamorphic rocks.
**Area 2**	Massam Glacier/ Garden Spur	S 84.54°–84.56°<br>W 174.91°–175.01°	Leo Sancho (2011)	**Garden Spur** is a narrow rocky ridge at the lowest end of Shackleton Glacier. The substrate is predominantly made up of crystalline plutonic (granite) or metamorphic rocks.
**Area 3**	Mt. Kyffin, The Gateway, Mt. Harcourt	S 83.49°–83.83°<br>E 170.79°–172.76°	Leo Sancho (2011)<br>Roman Türk (2003)	**The investigated area of Mt. Kyffin, Gateway, Mt. Harcourt** and surroundings is located at the southern edge of Beardsmore Glacier. The mountains are formed by Goldie Formation greywacke[1] and schist as well as crystalline plutonic (granite) or metamorphic rocks.
**Area 4a**	Darwin Area: Diamond Hills, Brown Hills	S 79.84°–79.88°<br>E 159.22°–159.39°	RomanTürk (2004, 2009)	**Diamond Hill** is located at the eastern edge of the Transantarctic Mountains, close to the Ross Ice Shelf and north from the Darwin Glacier. Climate conditions are characterized by higher air humidity and precipitation that support a higher diversity and abundance of lichens.<br>The **Brown Hills** are located in the north of Darwin Glacier. The Carlyon Granodiorite makes up most of the Brown Hills and includes a variably foliated, biotite-hornblende granodiorite and granite[2]. This site appears to be a particularly dry part of the continental Transantarctic Range.
**Area 4b**	Darwin Area: Bartrum Basin, Smith Valley, Lake Wellman	S 79.75°–79.95°<br>E 156.70°–158.67°	RomanTürk (2004, 2007, 2009)	**Bartrum Basin** is a very dry area, located in the north-west of the Brown Hills very dry area. The dominant rock types are dolerite and granite.<br>The surroundings of the **Smith Valley** and **Lake Wellman** are characterized by a very dry climate, caused by a high evaporation rate due to low average air humidity and/or continuous winds originating from the cold glacier regions. The bedrock surrounding this area is sandstone from the Beacon Group and dolerite from the Ferrar dolerite sills.
**Area 5**	McMurdo Dry Valleys	S 78.02°–78.17°<br>E 163.62°–164.10°	Roman Türk (2010)<br>Ulrike Ruprecht (2009, 2011)	The landscape of the **McMurdo Dry Valleys** is a mosaic of glacially formed valleys with intervening high ground, ice-covered lackes, ephemeral streams, arid rocky soils, ice-cemented soils, and surrounding glaciers along the steep scree and boulder slopes[3-5]. There are four main valleys (Miers Valley, Garwood Valley, Hidden Valley, and Marshall Valley) and some other extensive ice-free areas (Shangri-La). The valleys have the typical glaciated form with a U-cross-section with steep sides, often with scree slopes, and the valley floors are covered with glacial drift.

Supplementary Table S2. Samples used in this study, with information on collecting localities and Genbank accession numbers of different markers.

				**Mycobiont**				**Associated green micro algae (*Trebouxia*)**			
					**Accession numbers**				**Accession numbers**		
**Voucher ID**	**Area**	**Latitude**	**Longitude**	**Species name**	**nrITS**	**mtSSU**	***RPB1***	**OTU ID**	**nrITS2**	**psbJ-L**	**COX2**
MAF_DP1_01	1	-85.539	-151.150	*Lecidea cancriformis* Dodge & Baker	MK208709	MK205016	MK226962	*Tr*_S02	MK226844	MK226760	MK227045
MAF_DP1_02	1	-85.539	-151.150	*Lecanora fuscobrunnea* Dodge & Baker	MK208710	MK205017	MK226963	*Tr*_S02	MK226845	MK226761	MK227046
MAF_DP1_04	1	-85.539	-151.150	*Lecidea cancriformis* Dodge & Baker	MK208711	MK205018	MK226964	*Tr*_S02	MK226846	MK226800	MK227047
MAF_DP1_06	1	-85.539	-151.150	*Lecidea cancriformis* Dodge & Baker	MK208712	MK205019	MK226965	*Tr*_S02	-	MK226812	MK227048
MAF_DP1_07	1	-85.539	-151.150	*Lecanora* sp. 3	MK208713	-	-	*Tr*_A02	MK226847	MK226736	MK227049
MAF_DP1_08	1	-85.539	-151.150	*Lecanora fuscobrunnea* Dodge & Baker	MK208714	MK205021	MK226966	*Tr*_A02	MK226848	MK226737	MK227050
MAF_DP1_09	1	-85.539	-151.150	*Lecidea cancriformis* Dodge & Baker	MK208715	MK205022	-	*Tr*_S02	MK226849	MK226801	MK227051
MAF_DP1_10	1	-85.539	-151.150	*Lecidea cancriformis* Dodge & Baker	MK208716	MK205023	MK226967	*Tr*_S02	MK226850	MK226802	MK227052
MAF_DP1_11	1	-85.539	-151.150	*Lecidea cancriformis* Dodge & Baker	MK208717	MK205024	MK226968	*Tr*_S02	MK226851	MK226762	MK227053
MAF_DP1_15	1	-85.539	-151.150	*Lecidella greenii* Ruprecht & Türk	MK208718	-	MK226969	*Tr*_A02	MK226852	MK226738	MK227054
MAF_DP1_19	1	-85.539	-151.150	*Lecidea cancriformis* Dodge & Baker	MK208719	MK205025	MK226970	-	-	-	-
MAF_DP1_20	1	-85.539	-151.150	*Lecanora fuscobrunnea* Dodge & Baker	MK208720	MK205026	MK226971	*Tr*_A02	MK226853	MK226739	MK227055
MAF_DP1_22	1	-85.539	-151.150	*Lecidella siplei* Dodge & Baker	MK208721	-	-	*Tr*_A02	MK226854	MK226740	MK227056
MAF_DP1_24	1	-85.539	-151.150	*Lecidea cancriformis* Dodge & Baker	MK208722	MK205027	MK226972	*Tr*_I01	MK226855	-	-
MAF_DP1_25	1	-85.539	-151.150	*Lecidea cancriformis* Dodge & Baker	MK208723	MK205028	MK226973	*Tr*_A02	MK226856	MK226741	MK227057
MAF_DP1_28	1	-85.539	-151.150	*Lecanora fuscobrunnea* Dodge & Baker	MK208724	MK205029	MK226974	*Tr*_S02	MK226857	MK226771	MK227058
MAF_DP1_30	1	-85.539	-151.150	*Lecidea cancriformis* Dodge & Baker	MK208725	MK205030	-	*Tr*_S02	MK226858	MK226764	MK227059
MAF_DP1_32	1	-85.539	-151.150	*Lecanora fuscobrunnea* Dodge & Baker	MK208726	MK205031	MK226975	*Tr*_A02	MK226859	MK226742	MK227060
MAF_DP1_33	1	-85.539	-151.150	*Lecidea cancriformis* Dodge & Baker	MK208727	MK205032	MK226976	*Tr*_S02	MK226860	MK226765	MK227061
MAF_DP1_34	1	-85.539	-151.150	*Lecidea cancriformis* Dodge & Baker	MK208728	MK205033	MK226977	Tr_S02	MK226861	MK226813	MK227062
MAF_DP1_35	1	-85.539	-151.150	*Lecidella siplei* Dodge & Baker	MK208729	-	-	*Tr*_A02	MK226862	MK226743	MK227063
MAF_DP1_36	1	-85.539	-151.150	*Lecidea cancriformis* Dodge & Baker	MK208730	MK205034	MK226978	*Tr*_S02	MK226863	MK226815	MK227064
MAF_DP1_39	1	-85.539	-151.150	*Lecidea cancriformis* Dodge & Baker	MK208731	MK205035	MK226979	*Tr*_S02	-	MK226816	MK227065
MAF_DP1_50	1	-85.539	-151.150	*Lecanora fuscobrunnea* Dodge & Baker	MK208732	MK205036	MK226981	*Tr*_A02	-	MK226745	MK227067
MAF_DP1_51	1	-85.539	-151.150	*Lecidella greenii* Ruprecht & Türk	MK208733	MK205037	MK226982	*Tr*_A02	MK226865	MK226746	MK227068
MAF_DP1_52	1	-85.539	-151.150	*Carbonea* sp. URm1	MK208734	MK205038	-	*Tr*_A02	MK226866	MK226747	MK227069
MAF_DP1_54	1	-85.539	-151.150	*Carbonea* sp. 2	MK208735	-	MK226984	*Tr*_S02	MK226868	MK226772	MK227071
MAF_DP1_57	1	-85.539	-151.150	*Lecidella siplei* Dodge & Baker	MK208736	-	-	*Tr*_A02	MK226869	-	MK227072
MAF_GR1_29	3	-83.487	170.790	*Lecidea cancriformis* Dodge & Baker	MK208737	MK205039	MK226985	-	-	-	-
MAF_GS1_12	2	-84.535	-174.954	*Lecidea andersonii* Filson	MK208738	MK205040	-	*Tr*_S02	MK226871	MK226763	MK227075
MAF_GS1_13	2	-84.535	-174.954	*Lecidella siplei* Dodge & Baker	MK208739	-	-	*Tr*_A02	MK226872	-	MK227076

				**Mycobiont**				**Associated green micro algae (*Trebouxia*)**			
					**Accession numbers**				**Accession numbers**		
**Voucher ID**	**Area**	**Latitude**	**Longitude**	**Species name**	**nrITS**	**mtSSU**	***RPB1***	**OTU ID**	**nrITS2**	**psbJ-L**	**COX2**
MAF_GS1_44	2	-84.535	-174,954	*Lecidea andersonii* Filson	MK208740	MK205041	-	*Tr*_A02	MK226873	-	MK227077
MAF_GS1_45	2	-84.535	-174.954	*Lecanora physciella* (Darb.) Hertel	MK208741	-	MK226986	*Tr*_S02	MK226874	MK226766	MK227078
MAF_GS1_58	2	-84.535	-174.954	*Lecidella siplei* Dodge & Baker	MK208742	-	-	*Tr*_A02	MK226875	-	MK227079
MAF_GS1_60	2	-84.535	-174.954	*Lecidella siplei* Dodge & Baker	MK208743	-	-	*Tr*_A02	MK226876	-	-
MAF_GS1_61	2	-84.535	-174.954	*Lecidea andersonii* Filson	MK208744	MK205042	MK226987	*Tr*_A02	MK226877	MK226749	MK227080
MAF_GS1_62	2	-84.535	-174.954	*Lecidella siplei* Dodge & Baker	MK208745	-	-	*Tr*_A02	MK226878	MK226750	MK227081
MAF_GS1_64	2	-84.535	-174.954	*Lecidella* sp. nov2	MK208746	MK205043	MK226988	Tr_S02	MK226879	MK226775	MK227082
MAF_HS7_59	3	-83.806	172.262	*Lecidella* sp. nov2	MK208747	MK205044	MK226989	*Tr*_S02	MK226880	MK226810	MK227083
MAF_MG1_16	2	-84.559	-175.009	*Lecidea andersonii* Filson	MK208748	MK205045	MK226990	*Tr*_A02	MK226881	MK226751	MK227084
MAF_MG1_17	2	-84.559	-175.009	*Lecidea andersonii* Filson	MK208749	MK205046	-	*Tr*_A02	MK226882	MK226752	MK227085
MAF_MG1_23	2	-84.559	-175.009	*Lecidea andersonii* Filson	MK208750	MK205047	MK226991	*Tr*_A02	-	-	MK227086
MAF_MG1_47	2	-84.559	-175.009	*Lecidea cancriformis* Dodge & Baker	MK208751	-	-	*Tr*_I17	MK226883	-	-
MAF_MK1_03	3	-83.775	171.828	*Lecanora fuscobrunnea* Dodge & Baker	MK208752	MK205048	MK226992	*Tr*_A02	MK226884	MK226753	MK227087
MAF_MK1_18	3	-83.775	171.828	*Lecidella* sp. nov2	MK208753	-	-	*Tr*_S02	MK226886	MK226767	MK227089
MAF_MK1_21	3	-83.775	171.828	*Lecidella* sp. nov2	MK208754	MK205049	-	*Tr*_S02	MK226887	MK226788	MK227090
MAF_MK1_26	3	-83.775	171.828	*Lecidea cancriformis* Dodge & Baker	MK208755	MK205050	MK226993	*Tr*_S02	MK226888	MK226776	MK227091
MAF_MK1_27	3	-83.775	171.828	*Lecidella* sp. nov2	MK208756	MK205051	MK226994	*Tr*_S02	MK226889	MK226777	MK227092
MAF_MK1_31	3	-83.775	171.828	*Lecidea cancriformis* Dodge & Baker	MK208757	MK205052	MK226995	*Tr*_I01	MK226890	-	-
MAF_MK1_37	3	-83.775	171.828	*Lecidea cancriformis* Dodge & Baker	MK208758	MK205053	MK226996	*Tr*_I01	MK226891	-	-
MAF_MK1_38	3	-83.775	171.828	*Lecidea cancriformis* Dodge & Baker	MK208759	MK205054	-	*Tr*_S02	MK226892	MK226778	MK227093
MAF_MK1_40	3	-83.775	171.828	*Lecanora fuscobrunnea* Dodge & Baker	MK208760	MK205055	MK226997	*Tr*_A02	MK226893	MK226754	MK227094
MAF_MK1_43	3	-83.775	171.828	*Lecidella* sp. nov2	MK208761	MK205056	-	*Tr*_S02	MK226894	MK226779	MK227095
MAF_MK1_48	3	-83.775	171.828	*Lecidea cancriformis* Dodge & Baker	MK208762	MK205057	MK226998	*Tr*_S02	MK226895	MK226780	MK227096
MAF_MK1_49	3	-83.775	171.828	*Lecidella* sp. nov2	MK208763	MK205058	MK226999	*Tr*_S02	MK226896	MK226799	MK227097
MAF_MK1_55	3	-83.775	171.828	*Lecidea cancriformis* Dodge & Baker	MK208764	MK205059	MK227000	-	-	-	-
MAF_MK1_56	3	-83.775	171.828	*Lecanora fuscobrunnea* Dodge & Baker	MK208765	MK205060	MK227001	*Tr*_S02	MK226897	MK226781	MK227098
MAF_MK1_63	3	-83.775	171.828	*Lecanora physciella* (Darb.) Hertel	MK208766	MK205061	MK227002	*Tr*_S02	MK226898	MK226789	MK227099
MAF_Sancho	3	-83.761	172.755	*Lecidea cancriformis* Dodge & Baker	GU074439	GU074489	MK227003	*Tr*_A04a	JN204838	-	-
MAF_Sancho	3	-83.761	172.755	*Carbonea* sp. 2	MK208767	-	-	*Tr*_A04a	JN204839	-	-
T33335	3	-83.803	172.207	*Lecidella* sp. nov2	MK208768	MK205062	-	*Tr*_S18	MK226899	-	-
T33338	3	-83.803	172.207	*Lecidella* sp. nov2	MK208769	MK205063	-	*Tr*_S02	MK226900	-	-
T33346	3	-83.803	172.207	*Lecanora physciella* (Darb.) Hertel	JN873878	-	-	-	-	-	-
T33348	3	-83.803	172.207	*Lecidea cancriformis* Dodge & Baker	-	MK205064	-	-	-	-	-

				**Mycobiont**				**Associated green micro algae (*Trebouxia*)**			
					**Accession numbers**				**Accession numbers**		
**Voucher ID**	**Area**	**Latitude**	**Longitude**	**Species name**	**nrITS**	**mtSSU**	***RPB1***	**OTU ID**	**nrITS2**	**psbJ-L**	**COX2**
T33446	3	-83.828	172.749	*Lecidea cancriformis* Dodge & Baker	-	MK205065	-	*Tr*_S18	MK226901	-	-
T33449	3	-83.761	172.755	*Lecidella siplei* Dodge & Baker	JN873897	-	-	*Tr*_A02	JN204729	-	-
T33456	3	-83.761	172.755	*Lecidea andersonii* Filson	-	MK205066	MK227004	*Tr*_A02	-	-	MK227100
T33457	3	-83.761	172.755	*Lecidella siplei* Dodge & Baker	JN873898	-	-	*Tr*_A02	JN204731	-	-
T35540	4a	-79.842	159.363	*Lecidella siplei* Dodge & Baker	MK208770	-	-	-	-	-	-
T35544	4a	-79.838	159.341	*Lecanora fuscobrunnea* Dodge & Baker	JN873873	-	-	-	-	-	-
T35559	4a	-79.838	159.221	*Lecidea cancriformis* Dodge & Baker	MK208771	MK205067	MK227005	*Tr*_S18	-	MK285375	-
T35604	4a	-79.836	159.317	*Lecidea cancriformis* Dodge & Baker	EU257671	GU074480	MK227006	*Tr*_S18	JN204749	-	-
T35620	4a	-79.835	159.385	*Lecidea cancriformis* Dodge & Baker	EU257672	-	-	*Tr*_I01	JN204750	-	-
T35622	4a	-79.835	159.392	*Lecidea cancriformis* Dodge & Baker	MK208772	MK205068	MK227007	-	-	-	-
T35647	4a	-79.851	159.341	*Carbonea* sp. 2	JN873866	-	-	*Tr*_S02	JN204751	-	-
T35650	4a	-79.851	159.341	*Carbonea* sp. 2	JN873867	-	MK227008	*Tr*_S18	JN204752	-	-
T35662	4a	-79.840	159.332	*Lecidea cancriformis* Dodge & Baker	EU257673	-	-	*Tr*_I01	JN204753	-	-
T35664	4a	-79.851	159.341	*Lecanora fuscobrunnea* Dodge & Baker	JN873874	-	-	-	-	-	-
T35686	4a	-79.842	159.363	*Carbonea* sp. 2	JN873868	-	-	-	-	-	-
T42988	4b	-79.889	156.764	*Lecidea cancriformis* Dodge & Baker	GU074435	GU074481	MK227009	*Tr*_S18	MK226902	MK226818	MK227101
T42990	4b	-79.926	156.890	*Lecidea cancriformis* Dodge & Baker	GU170841	-	MK227010	*Tr*_S18	JN204770	MK226819	MK227102
T42991	4b	-79.917	156.759	*Lecidea cancriformis* Dodge & Baker	GU170842	MK205069	MK227011	*Tr*_S18	MK226903	-	-
T42992	4b	-79.917	156.751	*Lecidea cancriformis* Dodge & Baker	GU074436	GU074482	MK227012	*Tr*_S18	JN204771	MK226820	MK227103
T42994	4b	-79.879	157.539	*Lecanora fuscobrunnea* Dodge & Baker	GU170839	MK205070	MK227013	*Tr*_S18	MK226904	-	-
T44625	4a	-79.868	159.360	*Lecanora fuscobrunnea* Dodge & Baker	MK208773	MK205071	MK227014	*Tr*_A02	MK226905	MK226755	MK227104
T44626	4a	-79.882	159.361	*Carbonea* sp. URm1	JN873865	-	MK227015	*Tr*_I01	JN204797	-	-
T44628	4a	-79.865	159.352	*Lecanora fuscobrunnea* Dodge & Baker	JN873875	MK205072	MK227016	*Tr*_S02	-	MK226804	MK227105
T44632	4a	-79.868	159.352	*Lecanora fuscobrunnea* Dodge & Baker	MK208774	MK205073	MK227017	*Tr*_I01	JN204800	-	-
T44633	4a	-79.868	159.358	*Lecidea cancriformis* Dodge & Baker	MK208775	MK205074	-	*Tr*_S02	-	MK226817	MK227106
T44634	4a	-79.869	159.341	*Lecidea cancriformis* Dodge & Baker	GU074434	GU074486	MK227018	*Tr*_S18	JN204801	KF907601	-
T44636	4a	-79.869	159.358	*Lecidella greenii* Ruprecht & Türk	MK208776	-	-	*Tr*_A02	MK226906	-	-
T44638	4a	-79.869	159.358	*Lecidea cancriformis* Dodge & Baker	-	MK205075	-	*Tr*_S18	MK226907	MK226827	MK227107
T44640	4a	-79.869	159.358	*Lecidea cancriformis* Dodge & Baker	MK208777	MK205076	MK227019	*Tr*_S02	MK226908	MK226809	MK227108
T44641	4a	-79.866	159.365	*Carbonea* sp. 2	JN873871	-	MK227020	*Tr*_A04a	JN204803	KF907602	-
T44643	4a	-79.859	159.238	*Lecidea cancriformis* Dodge & Baker	MK208778	MK205077	-	*Tr*_S18	-	MK226831	MK227109
T44645	4a	-79.857	159.283	*Lecidea cancriformis* Dodge & Baker	MK208779	MK205078	MK227021	*Tr*_S02	MK226909	MK226790	MK227110
T44646	4a	-79.857	159.283	*Lecidea cancriformis* Dodge & Baker	MK208780	MK205079	-	*Tr*_S02	MK226910	MK226793	MK227111

				**Mycobiont**				**Associated green micro algae (*Trebouxia*)**			
					**Accession numbers**				**Accession numbers**		
**Voucher ID**	**Area**	**Latitude**	**Longitude**	**Species name**	**nrITS**	**mtSSU**	***RPB1***	**OTU ID**	**nrITS2**	**psbJ-L**	**COX2**
T44647	4a	-79.858	159.288	*Lecidea cancriformis* Dodge & Baker	MK208781	MK205080	-	*Tr*_S02	MK226911	MK226794	MK227112
T44648	4a	-79.858	159.288	*Lecidea cancriformis* Dodge & Baker	MK208782	MK205081	-	-	-	-	-
T44649	4a	-79.858	159.288	*Lecidea cancriformis* Dodge & Baker	MK208783	MK205082	-	*Tr*_S02	MK226912	MK226791	MK227113
T44650	4a	-79.858	159.288	*Lecanora fuscobrunnea* Dodge & Baker	MK208784	MK205083	MK227022	*Tr*_I01	MK285376	-	-
T44651	4a	-79.858	159.288	*Lecidea cancriformis* Dodge & Baker	MK208785	MK205084	-	*Tr*_S02	MK226913	MK226795	MK227114
T44652	4a	-79.858	159.288	*Lecidea cancriformis* Dodge & Baker	MK208786	MK205085	-	*Tr*_S02	MK226914	MK226796	MK227115
T44655	4a	-79.874	159.339	*Carbonea* sp. 2	MK208787	-	MK227023	*Tr*_S02	MK226915	MK226805	MK227116
T44656	4a	-79.863	159.373	*Lecidea cancriformis* Dodge & Baker	MK208788	MK205086	-	*Tr*_S02	MK226916	MK226797	MK227117
T44657	4a	-79.863	159.373	*Lecanora fuscobrunnea* Dodge & Baker	JN873876	MK205087	MK227024	*Tr*_S02	JN204804	MK226806	MK227118
T44659	4a	-79.864	159.368	*Lecidea cancriformis* Dodge & Baker	MK208789	MK205088	-	*Tr*_S02	MK226917	MK226798	MK227119
T44665	4a	-79.869	159.345	*Lecidea cancriformis* Dodge & Baker	MK208790	MK205089	-	*Tr*_S02	MK226918	MK226814	MK227120
T44666	4a	-79.869	159.345	*Lecidea cancriformis* Dodge & Baker	MK208791	MK205090	-	-	-	-	-
T44667	4a	-79.869	159.345	*Lecidea cancriformis* Dodge & Baker	MK208792	MK205091	MK227025	*Tr*_S02	MK226919	MK226782	MK227121
T44669	4a	-79.863	159.378	*Lecidea cancriformis* Dodge & Baker	MK208793	-	-	*Tr*_S02	MK226920	MK226768	MK227122
T44670	4a	-79.863	159.378	*Lecidea cancriformis* Dodge & Baker	MK208794	MK205092	-	Tr_I17	MK226921	-	-
T44674	4a	-79.877	159.326	*Lecidea cancriformis* Dodge & Baker	MK208795	-	-	Tr_S02	MK226923	MK226783	MK227124
T44675	4a	-79.877	159.326	*Lecanora fuscobrunnea* Dodge & Baker	-	MK205093	-	-	JN204806	-	-
T44676	4a	-79.883	159.348	*Lecidea cancriformis* Dodge & Baker	MK208796	MK205094	MK227026	*Tr*_S18	MK226924	MK226832	MK227125
T44677	4a	-79.883	159.348	*Lecidea cancriformis* Dodge & Baker	MK208797	MK205095	MK227027	*Tr*_A02	MK226925	MK226756	MK227126
T44679	4a	-79.877	159.331	*Lecidea cancriformis* Dodge & Baker	MK208798	MK205096	-	*Tr*_S02	MK226927	MK226769	MK227128
T44687	4a	-79.863	159.379	*Lecanora fuscobrunnea* Dodge & Baker	MK208799	-	-	-	-	-	MK227130
T44688	4a	-79.863	159.382	*Lecanora fuscobrunnea* Dodge & Baker	JN873877	MK205097	MK227028	*Tr*_S02	JN204807	MK226807	MK227131
T44690	4a	-79.863	159.382	*Lecidea cancriformis* Dodge & Baker	MK208800	MK205098	-	*Tr*_S02	MK226930	MK226786	MK227133
T44692	4a	-79.866	159.366	Lecidea *cancriformis* Dodge & Baker	MK208801	MK205099	MK227029	*Tr*_S02	JN204809	KF907603	-
T44694	4b	-79.755	158.503	*Lecidea cancriformis* Dodge & Baker	MK208802	MK205100	-	*Tr*_I01	MK226931	-	-
T44695	4b	-79.761	158.503	*Lecanora fuscobrunnea* Dodge & Baker	MK208803	MK205101	MK227030	-	-	-	-
T44697	4b	-79.758	158.598	*Lecanora fuscobrunnea* Dodge & Baker	MK208804	MK205102	-	*Tr*_S02	MK226932	MK226770	MK227134
T44698	4b	-79.755	158.627	*Lecidea cancriformis* Dodge & Baker	MK208805	MK205103	MK227031	*Tr*_S18	MK226933	MK226825	MK227135
T44699	4b	-79.762	158.636	*Lecanora fuscobrunnea* Dodge & Baker	MK208806	MK205104	MK227032	*Tr*_A02	MK226934	MK226758	MK227136
T44700	4b	-79.762	158.637	*Lecanora fuscobrunnea* Dodge & Baker	MK208807	MK205105	MK227033	*Tr*_I01	MK226935	-	-
T44701	4b	-79.757	158.608	*Lecidea cancriformis* Dodge & Baker	MK208808	MK205106	-	*Tr*_S18	MK226936	MK226833	MK227137
T44702	4b	-79.756	158.610	*Lecanora fuscobrunnea* Dodge & Baker	MK208809	MK205107	MK227034	*Tr*_A02	MK226937	MK226759	MK227138
T44703	4b	-79.756	158.614	*Lecidea cancriformis* Dodge & Baker	-	MK205108	MK227035	*Tr*_S18	MK226938	MK226828	MK227139

				Mycobiont				Associated green micro algae (*Trebouxia*)			
					Accession numbers				Accession numbers		
Voucher ID	Area	Latitude	Longitude	Species name	nrITS	mtSSU	*RPB1*	OTU ID	nrITS2	psbJ-L	COX2
T44704	4b	-79.756	158.617	*Lecidea cancriformis* Dodge & Baker	-	MK205109	-	*Tr*_S18	MK226939	MK226829	MK227140
T44705	4b	-79.756	158.611	*Lecanora fuscobrunnea* Dodge & Baker	MK208810	-	-	*Tr*_S02	MK226940	MK226811	MK227141
T44707	4b	-79.758	158.598	*Lecidea cancriformis* Dodge & Baker	MK208811	MK205110	MK227036	*Tr*_S18	MK226941	MK226834	MK227142
T44708	4b	-79.753	158.549	*Lecidea cancriformis* Dodge & Baker	MK208812	MK205111	-	*Tr*_S18	-	MK226823	MK227143
T44709	4b	-79.753	158.541	*Lecidea cancriformis* Dodge & Baker	MK208813	MK205112	MK227037	*Tr*_S18	MK226942	MK226826	MK227144
T44712	4b	-79.759	158.507	*Lecidea cancriformis* Dodge & Baker	GU074438	GU074487	MK227038	*Tr*_S18	JN204811	-	-
T44713	4b	-79.759	158.511	*Lecidea cancriformis* Dodge & Baker	MK208814	-	-	*Tr*_S18	MK226945	MK226836	MK227147
T44714	4b	-79.763	158.497	*Lecidea cancriformis* Dodge & Baker	MK208815	MK205113	-	*Tr*_S18	MK226946	MK226830	MK227148
T44715	4b	-79.758	158.602	*Lecidea cancriformis* Dodge & Baker	-	MK205114	MK227039	*Tr*_S18	MK226947	MK226837	MK227149
T44716	4b	-79.758	158.606	*Lecidea cancriformis* Dodge & Baker	-	MK205115	MK227040	*Tr*_S18	MK226948	MK226841	MK227150
T44717	4b	-79.755	158.620	*Lecanora fuscobrunnea* Dodge & Baker	MK208816	MK205116	MK227041	*Tr*_S02	MK226949	MK226787	MK227151
T44719	4b	-79.924	156.814	*Lecanora fuscobrunnea* Dodge & Baker	MK208817	MK205117	MK227042	*Tr*_S18	MK226950	MK226821	MK227152
T44720	4b	-79.949	156.789	*Lecanora fuscobrunnea* Dodge & Baker	GU170840	MK205118	MK227043	*Tr*_S02	MK226951	MK226808	MK227153
T44721	4b	-79.754	158.636	*Lecidea cancriformis* Dodge & Baker	MK208818	MK205119	MK227044	*Tr*_S18	MK226952	MK226842	MK227154
T44723	4b	-79.878	157.527	*Lecidea cancriformis* Dodge & Baker	MK208819	MK205120	-	*Tr*_S18	MK226953	MK226838	MK227155
T44727	4b	-79.892	157.524	*Lecidea cancriformis* Dodge & Baker	GU074437	GU074488	-	*Tr*_S18	JN204812	-	-
T44787	4b	-79.929	156.705	*Lecidea cancriformis* Dodge & Baker	MK208820	MK205122	-	*Tr*_S18	MK226955	MK226839	MK227157

Supplementary Table S3. Additional samples taken from Perez-Ortega et al. (2012)[6] and used in this study, with information on collecting localities and Genbank accession numbers.

				Mycobiont		Associated green micro algae *(Trebouxia)*	
Laboratory code	Area	Latitude	Longitude	Species name	Accession numbers nrITS	OTU ID	Accession numbers nrITS
s106	5	-78.113	163.782	*Lecanora* sp. 2	JX036037	*Tr*_A02	JX036159
s113	5	-78.114	163.854	*Lecidea cancriformis* Dodge & Baker	JX036044	*Tr*_S15	JX036166
s114	5	-78.114	163.854	*Lecidea cancriformis* Dodge & Baker	JX036045	*Tr*_A02	JX036167
s115	5	-78.066	163.870	*Lecidella greenii* Ruprecht & Türk	JX036046	*Tr*_A02	JX036168
s120	5	-78.083	163.768	*Lecidea polypycnidophora* Ruprecht & Türk	JX036051	*Tr*_A02	JX036172
s121	5	-78.024	163.900	*Carbonea vorticosa* (Flörke) Hertel	JX036052	*Tr*_A02	JX036173
s122	5	-78.110	163.787	*Carbonea vorticosa* (Flörke) Hertel	JX036053	*Tr*_A02	JX036174
s123	5	-78.110	163.787	*Carbonea vorticosa* (Flörke) Hertel	JX036054	*Tr*_A02	JX036175

				Mycobiont		Associated green micro algae (*Trebouxia*)	
**Laboratory code**	**Area**	**Latitude**	**Longitude**	**Species name**	**Accession numbers nrITS**	**OTU ID**	**Accession numbers nrITS**
s124	5	-78.057	163.844	*Rhizoplaca macleanii* (Dodge) Castello	JX036055	*Tr*_A02	JX036176
s125	5	-78.057	163.844	*Rhizoplaca macleanii* (Dodge) Castello	JX036056	*Tr*_A02	JX036177
s171	5	-78.036	163.837	*Lecanora* sp. 2	JX036076	*Tr*_A02	JX036197
s173	5	-78.114	163.854	*Lecanora* sp. 2	JX036078	*Tr*_A02	JX036199
s175	5	-78.033	163.849	*Lecanora* sp. 2	JX036080	*Tr*_A02	JX036201
s179	5	-78.063	163.809	*Lecanora* sp. 2	JX036084	*Tr*_A02	JX036205
s181	5	-78.111	163.858	*Lecidella greenii* Ruprecht & Türk	JX036086	*Tr*_A02	JX036207
s190	5	-78.025	163.899	*Lecanora* sp. 3	JX036095	*Tr*_A02	JX036216
s191	5	-78.025	163.900	*Lecanora* sp. 3	JX036096	*Tr*_A02	JX036217
s192	5	-78.030	163.834	*Lecidella greenii* Ruprecht & Türk	JX036097	*Tr*_A02	JX036218
s197	5	-78.061	163.791	*Rhizoplaca macleanii* (Dodge) Castello	JX036101	*Tr*_A02	JX036222
s198	5	-78.061	163.791	*Rhizoplaca macleanii* (Dodge) Castello	JX036102	*Tr*_A02	JX036223
s201	5	-78.024	163.900	*Rhizoplaca macleanii* (Dodge) Castello	JX036105	*Tr*_A02	JX036226
s202	5	-78.027	163.839	*Lecanora* cf. *mons-nivis* Darbishire	JX036106	*Tr*_A02	JX036227
s203	5	-78.027	163.839	*Lecidella greenii* Ruprecht & Türk	JX036107	*Tr*_A02	JX036228
s205	5	-78.068	163.861	*Rhizoplaca macleanii* (Dodge) Castello	JX036108	*Tr*_A02	JX036229
s206	5	-78.068	163.861	*Rhizoplaca macleanii* (Dodge) Castello	JX036109	*Tr*_A02	JX036230
s207	5	-78.068	163.861	*Rhizoplaca macleanii* (Dodge) Castello	JX036110	*Tr*_A02	JX036231
s208	5	-78.068	163.861	*Rhizoplaca macleanii* (Dodge) Castello	JX036111	*Tr*_A02	JX036232
s209	5	-78.068	163.861	*Rhizoplaca macleanii* (Dodge) Castello	JX036112	*Tr*_A02	JX036233
s212	5	-78.034	163.845	*Rhizoplaca macleanii* (Dodge) Castello	JX036115	*Tr*_A02	JX036236
s213	5	-78.034	163.845	*Rhizoplaca macleanii* (Dodge) Castello	JX036116	*Tr*_A02	JX036237
s214	5	-78.034	163.845	*Rhizoplaca macleanii* (Dodge) Castello	JX036117	*Tr*_A02	JX036238
s215	5	-78.070	163.711	*Lecanora fuscobrunnea* Dodge & Baker	JX036118	*Tr*_A02	JX036239
s230	5	-78.034	163.845	*Lecidella greenii* Ruprecht & Türk	JX036133	*Tr*_A02	JX036252
s232	5	-78.034	163.845	*Rhizoplaca macleanii* (Dodge) Castello	JX036135	*Tr*_A02	JX036254
s233	5	-78.034	163.845	*Rhizoplaca macleanii* (Dodge) Castello	JX036136	*Tr*_A02	JX036255
s235	5	-78.113	163.778	*Rhizoplaca macleanii* (Dodge) Castello	JX036138	*Tr*_A02	JX036257
s236	5	-78.113	163.778	*Rhizoplaca macleanii* (Dodge) Castello	JX036139	*Tr*_A02	JX036258
s237	5	-78.113	163.778	*Rhizoplaca macleanii* (Dodge) Castello	JX036140	*Tr*_A02	JX036259
s266	5	-78.075	163.791	*Lecidella greenii* Ruprecht & Türk	JX036141	*Tr*_A02	JX036260
s271	5	-78.047	164.104	*Rhizoplaca macleanii* (Dodge) Castello	JX036145	*Tr*_A02	JX036264
s272	5	-78.035	163.978	*Rhizoplaca macleanii* (Dodge) Castello	JX036146	*Tr*_A02	JX036265
s273	5	-78.030	163.949	*Rhizoplaca macleanii* (Dodge) Castello	JX036147	*Tr*_A02	JX036266
s274	5	-78.036	163.990	*Rhizoplaca macleanii* (Dodge) Castello	JX036148	*Tr*_A02	JX036267

				Mycobiont		Associated green micro algae (*Trebouxia*)	
Laboratory code	Area	Latitude	Longitude	Species name	Accession numbers nrITS	OTU ID	Accession numbers nrITS
s300	5	-78.030	163.834	*Lecidella greenii* Ruprecht & Türk	JX036150	*Tr*_A02	JX036269
s301	5	-78.030	163.834	*Lecidella greenii* Ruprecht & Türk	JX036151	*Tr*_A02	JX036270
s95	5	-78.024	163.900	*Rhizoplaca macleanii* (Dodge) Castello	JX036152	*Tr*_A02	JX036271

Supplementary Table S4. Additional samples taken from Wagner et al. (2020)[7] and used in this study, with information on collecting localities and Genbank accession numbers.

				Mycobiont				Associated green micro algae (*Trebouxia*)			
					Accession numbers				Accession numbers		
Voucher ID	Area	Latitude	Longitude	Species name	nrITS	mtSSU	*RPB1*	OTU ID	nrITS	psbJ-L	COX2
T46643	5	-78.031	163.865	*Rhizoplaca macleanii* (Dodge) Castello	MK970663	-	-	*Tr*_A02	MK970698	-	-
T46647b	5	-78.033	163.898	*Rhizoplaca macleanii* (Dodge) Castello	MK970665	MN023039	MN023053	*Tr*_A02	MK970698	-	-
T46651	5	-78.028	163.851	*Carbonea vorticosa* (Flörke) Hertel	MK970656	-	-	-	-	-	-
T46659	5	-78.023	163.903	*Lecidella greenii* Ruprecht & Türk	MK970671	-	MN023055	*Tr*_A02	MK970698	-	-
T46672	5	-78.024	163.898	*Rhizoplaca macleanii* (Dodge) Castello	MK970663	-	-	*Tr*_A02	MK970698	-	-
T46673	5	-78.027	163.851	*Rhizoplaca macleanii* (Dodge) Castello	MK970663	-	-	*Tr*_A02	MK970699	-	-
T46676	5	-78.028	163.851	*Lecidella greenii* Ruprecht & Türk	MK970671	-	-	*Tr*_A02	MK970699	-	-
T46677	5	-78.027	163.848	*Lecidea cancriformis* Dodge & Baker	MK970681	-	-	*Tr*_A02	MK970696	-	-
T46678	5	-78.028	163.850	*Rhizoplaca macleanii* (Dodge) Castello	MK970669	-	-	*Tr*_A02	MK970699	-	-
T46679	5	-78.036	163.971	*Rhizoplaca macleanii* (Dodge) Castello	MK970664	MN023039	-	*Tr*_A02	MK970696	-	-
T46680	5	-78.036	163.971	*Lecidea polypycnidophora* Ruprecht & Türk	MK970663	MN023043	MN023053	*Tr*_A02	MK970699	-	-
T46681	5	-78.032	163.951	*Rhizoplaca macleanii* (Dodge) Castello	MK970663	-	-	*Tr*_A02	MK970698	-	-
T46684	5	-78.044	163.986	*Lecidea cancriformis* Dodge & Baker	MK970677	-	-	*Tr*_S02	MK970693	-	-
T46685	5	-78.044	163.986	*Lecidea cancriformis* Dodge & Baker	MK970677	-	MN023056	*Tr*_S02	MK970693	-	MN023030
T46701	5	-78.020	163.805	*Rhizoplaca macleanii* (Dodge) Castello	MK970663	MN023034	-	*Tr*_A02	MK970698	-	-
T46706	5	-78.028	163.821	*Lecidella greenii* Ruprecht & Türk	MK970671	-	MN023054	*Tr*_A02	MK970698	-	-
T46710	5	-78.073	163.717	*Rhizoplaca macleanii* (Dodge) Castello	MK970666	-	-	*Tr*_A02	MK970698	-	-
T46713	5	-78.028	163.843	*Lecidea polypycnidophora* Ruprecht & Türk	MK970663	MN023043	MN023061	*Tr*_A02	MK970698	-	-
T46716	5	-78.040	163.802	*Lecidea polypycnidophora* Ruprecht & Türk	MK970663	MN023043	MN023061	*Tr*_A02	MK970698	-	-
T46717	5	-78.040	163.806	*Lecidea polypycnidophora* Ruprecht & Türk	MK970663	-	-	*Tr*_A02	MK970698	-	-
T46718	5	-78.040	163.807	*Carbonea vorticosa* (Flörke) Hertel	MK970656	-	-	*Tr*_A02	MK970698	-	-
T46719	5	-78.038	163.804	*Carbonea* sp. URm1	MK970657	-	-	*Tr*_A02	MK970698	-	-

				Mycobiont				Associated green micro algae (*Trebouxia*)			
					Accession numbers				Accession numbers		
**Voucher ID**	**Area**	**Latitude**	**Longitude**	**Species name**	**nrITS**	**mtSSU**	***RPB1***	**OTU ID**	**nrITS**	**psbJ-L**	**COX2**
T48769	5	-78.126	163.700	*Lecidella greenii* Ruprecht & Türk	MK970671	-	-	*Tr*_A02	MK970699	-	-
T48770	5	-78.127	163.690	*Lecanora* sp. 3	MK970659	-	-	*Tr*_A02	MK970701	-	-
T48773	5	-78.127	163.674	*Lecidea polypycnidophora* Ruprecht & Türk	MK970663	MN023043	-	*Tr*_A02	MK970699	MN023065	-
T48774	5	-78.135	163.626	*Rhizoplaca macleanii* (Dodge) Castello	MK970663	-	-	*Tr*_A02	MK970698	-	-
T48776	5	-78.165	163.753	*Lecidea cancriformis* Dodge & Baker	MK970679	MN023046	MN023057	*Tr*_A02	MK970702	-	-
T48777	5	-78.166	163.755	*Lecidea cancriformis* Dodge & Baker	MK970679	MN023046	MN023058	*Tr*_S15	MK970692	-	MN023031
T48778a	5	-78.149	163.769	*Rhizoplaca macleanii* (Dodge) Castello	MK970663	-	-	*Tr*_A02	MK970698	-	-
T48779	5	-78.164	163.755	*Rhizoplaca macleanii* (Dodge) Castello	MK970664	MN023039	-	*Tr*_A02	MK970696	-	-
T48781	5	-78.128	163.620	*Lecidea* sp. 6	MK620097	-	-	*Tr*_A02	MK970699	-	-
T48782	5	-78.123	163.642	*Lecidea cancriformis* Dodge & Baker	MK970679	MN023046	-	*Tr*_S18	MK970695	MN023070	MN023032
T48784	5	-78.121	163.683	Lecidea *polypycnidophora* Ruprecht & Türk	MK970663	MN023043	-	*Tr*_A02	MK970699	MN023065	-
T48785	5	-78.133	163.666	*Lecidella greenii* Ruprecht & Türk	MK970671	-	MN023054	*Tr*_S15	MK970692	-	-
T48787	5	-78.127	163.678	*Lecidella greenii* Ruprecht & Türk	MK970671	-	MN023054	*Tr*_A02	MK970699	-	-
T48788	5	-78.120	163.684	*Carbonea vorticosa* (Flörke) Hertel	MK970656	MN023033	MN023050	*Tr*_A02	MK970699	-	-
T48789	5	-78.152	163.739	*Carbonea* sp. 2	MK970654	-	MN023051	*Tr*_S15	MK970692	-	MN023031
T48790a	5	-78.120	163.682	*Carbonea vorticosa* (Flörke) Hertel	MK970656	MN023033	-	*Tr*_A02	MK970699	-	-
T48790b	5	-78.120	163.682	*Lecanora* sp. 3	MK970659	-	-	*Tr*_A02	MK970703	-	-
T48791a	5	-78.120	163.686	*Lecidella greenii* Ruprecht & Türk	MK970671	-	-	*Tr*_A02	MK970699	-	-
T48793a	5	-78.153	163.731	*Lecidea cancriformis* Dodge & Baker	MK970677	-	MN023056	*Tr*_S15	MK970692	-	MN023031
T48793b	5	-78.153	163.731	*Carbonea* sp. 2	MK970654	-	-	*Tr*_S15	MK970692	-	MN023031
T48794b	5	-78.151	163.735	*Rhizoplaca macleanii* (Dodge) Castello	MK970663	-	-	*Tr*_A02	MK970698	-	-
T48795a	5	-78.161	163.714	*Rhizoplaca macleanii* (Dodge) Castello	MK970668	-	-	*Tr*_A02	MK970698	-	-
T48797	5	-78.156	163.689	*Rhizoplaca macleanii* (Dodge) Castello	MK970667	-	-	*Tr*_A02	MK970698	-	-
T48798	5	-78.150	163.736	*Rhizoplaca macleanii* (Dodge) Castello	MK970667	-	-	*Tr*_A02	MK970698	-	-
T48799b	5	-78.145	163.620	*Lecidea cancriformis* Dodge & Baker	MK970679	-	-	*Tr*_A02	MK970698	-	-
T48799c	5	-78.145	163.620	*Lecidea cancriformis* Dodge & Baker	MK970677	-	-	*Tr*_S15	MK970692	-	-
T48800	5	-78.146	163.631	*Lecanora* sp. 2	MK970662	MN023036	MN023052	*Tr*_A02	MK970698	-	-
T48801a	5	-78.144	163.626	*Lecanora* sp. 3	MK970659	-	-	*Tr*_A02	MK970699	-	-
T48801c	5	-78.144	163.626	*Lecidella greenii* Ruprecht & Türk	MK970671	MN023040	-	*Tr*_A02	MK970699	-	-
T48803b	5	-78.148	163.630	*Carbonea* sp. URm1	MK970657	MN023034	-	*Tr*_A02	MK970699	-	-
T48804	5	-78.148	163.630	*Rhizoplaca macleanii* (Dodge) Castello	MK970670	-	MN023053	*Tr*_A02	MK970699	-	-
T48805	5	-78.144	163.626	*Lecidella greenii* Ruprecht & Türk	MK970671	-	-	*Tr*_A02	MK970698	-	-
T48806	5	-78.142	163.628	*Carbonea* sp. URm1	MK970657	MN023034	-	*Tr*_A02	MK970699	-	-

				**Mycobiont**				**Associated green micro algae (*Trebouxia*)**			
					**Accession numbers**				**Accession numbers**		
**Voucher ID**	**Area**	**Latitude**	**Longitude**	**Species name**	**nrITS**	**mtSSU**	***RPB1***	**OTU ID**	**nrITS**	**psbJ-L**	**COX2**
T48807	5	-78.141	163.631	*Lecidea andersonii* Filson	MK970673	MN023042	MN023060	*Tr*_A02	MK970702	-	-
T48809	5	-78.148	163.657	*Lecidella greenii* Ruprecht & Türk	MK970671	-	-	*Tr*_A02	MK970699	-	-
T48811a	5	-78.142	163.657	*Lecidella greenii* Ruprecht & Türk	MK970671	-	MN023055	*Tr*_A02	MK970698	-	-
T48812a	5	-78.138	163.619	*Lecidella greenii* Ruprecht & Türk	MK970671	-	-	*Tr*_A02	MK970699	-	-
T48812b	5	-78.138	163.619	*Lecidella greenii* Ruprecht & Türk	MK970671	-	MN023055	*Tr*_A02	MK970699	-	-
T48813	5	-78.142	163.657	*Lecidea* UCR1	MK970675	MN023044	-	*Tr*_A02	MK970699	-	-
T48817a	5	-78.113	163.785	*Rhizoplaca macleanii* (Dodge) Castello	MK970663	-	-	*Tr*_A02	MK970698	-	-
T48817b	5	-78.113	163.785	*Carbonea* sp. 2	MK970655	-	-	*Tr*_A02	MK970702	-	-
T48820	5	-78.114	163.780	*Carbonea* sp. 2	MK970654	-	-	*Tr*_A02	MK970698	-	-
T48821	5	-78.114	163.779	*Rhizoplaca macleanii* (Dodge) Castello	MK970665	-	-	*Tr*_A02	MK970698	-	-
T48823a	5	-78.098	163.710	*Carbonea vorticosa* (Flörke) Hertel	MK970656	-	-	-	-	-	-
T48825	5	-78.097	163.691	*Carbonea* sp. 2	MK970654	MN023035	-	*Tr*_A02	MK970699	-	-
T48826	5	-78.097	163.717	*Lecanora fuscobrunnea* Dodge & Baker	MK970661	-	-	*Tr*_S02	MK970694	MN023068	-
T48828a	5	-78.110	163.858	*Lecidella greenii* Ruprecht & Türk	MK970671	-	MN023054	-	-	-	-
T48828b	5	-78.110	163.858	*Lecidella greenii* Ruprecht & Türk	MK970671	-	-	*Tr*_A02	MK970698	-	-
T48829	5	-78.111	163.858	*Lecidella greenii* Ruprecht & Türk	MK970671	-	-	*Tr*_A02	MK970699	-	-
T48831	5	-78.111	163.858	*Carbonea vorticosa* (Flörke) Hertel	MK970656	MN023033	-	*Tr*_A02	MK970699	-	-
T48832	5	-78.112	163.824	*Carbonea* sp. 2	MK970654	-	MN023051	*Tr*_A02	MK970702	-	-
T48836	5	-78.099	163.778	*Lecanora* cf. *mons-nivis* Darbishire	MK970658	-	-	*Tr*_A02	MK970699	-	-
T48837	5	-78.098	163.777	*Lecidella greenii* Ruprecht & Türk	MK970671	-	MN023055	*Tr*_A02	MK970699	-	-
T48839	5	-78.114	163.854	*Lecidella greenii* Ruprecht & Türk	MK970671	-	-	*Tr*_A02	MK970699	-	-
T48841a	5	-78.068	163.861	*Rhizoplaca macleanii* (Dodge) Castello	MK970663	-	-	*Tr*_A02	MK970698	-	-
T48841b	5	-78.068	163.861	*Carbonea vorticosa* (Flörke) Hertel	MK970656	-	MN023050	*Tr*_A02	MK970698	-	-
T48843a	5	-78.066	163.870	*Lecidea cancriformis* Dodge & Baker	MK970677	-	-	*Tr*_S02	MK970693	-	-
T48843c	5	-78.066	163.870	*Lecidea cancriformis* Dodge & Baker	MK970678	-	-	-	-	-	-
T48843d	5	-78.066	163.870	*Rhizoplaca macleanii* (Dodge) Castello	MK970663	-	-	*Tr*_A02	MK970699	-	-
T48843e	5	-78.066	163.870	*Rhizoplaca macleanii* (Dodge) Castello	MK970663	-	-	*Tr*_A02	MK970702	-	-
T48844	5	-78.067	163.863	*Lecidella greenii* Ruprecht & Türk	MK970671	-	-	*Tr*_A02	MK970698	-	-
T48851	5	-78.074	163.793	*Lecanora fuscobrunnea* Dodge & Baker	MK970660	MN023037	-	*Tr*_A02	MK970698	-	-
T48855b	5	-78.036	163.837	*Lecidea cancriformis* Dodge & Baker	MK970680	MN023046	MN023059	*Tr*_S15	MK970692	-	MN023031
T48857a	5	-78.036	163.827	*Lecidea* sp. 6	MK970684	MN023045	-	*Tr*_A02	MK970699	-	-
T48858	5	-78.025	163.975	*Lecidella greenii* Ruprecht & Türk	MK970671	-	-	-	-	-	-
T48859	5	-78.025	163.986	*Lecidea polypycnidophora* Ruprecht & Türk	MK970663	-	MN023061	*Tr*_A02	MK970698	-	-

				Mycobiont				Associated green micro algae (*Trebouxia*)			
					Accession numbers				Accession numbers		
Voucher ID	Area	Latitude	Longitude	Species name	nrITS	mtSSU	*RPB1*	OTU ID	nrITS	psbJ-L	COX2
T48860b	5	-78.036	163.836	*Lecidea* sp. 6	MK970684	MN023045	MN023064	*Tr*_A02	MK970699	-	-
T48861	5	-78.024	163.892	*Lecanora* sp. 3	MK970659	-	-	*Tr*_A02	MK970699	-	-
T48862	5	-78.037	163.978	*Lecidea cancriformis* Dodge & Baker	MK970682	-	-	-	-	-	-
T48864	5	-78.039	163.989	*Lecidea cancriformis* Dodge & Baker	MK970679	-	-	*Tr*_A02	MK970702	-	-
T48865a	5	-78.036	163.990	*Rhizoplaca macleanii* (Dodge) Castello	MK970666	-	-	*Tr*_A02	MK970698	MN023067	-
T48867a	5	-78.043	164.104	*Rhizoplaca macleanii* (Dodge) Castello	MK970663	-	-	*Tr*_A02	MK970696	-	-
T48867c	5	-78.043	164.104	*Rhizoplaca macleanii* (Dodge) Castello	MK970666	-	-	*Tr*_A02	MK970702	-	-
T48869	5	-78.037	163.978	*Lecidella greenii* Ruprecht & Türk	MK970671	-	-	-	-	-	-
T48872	5	-78.030	163.951	*Lecidella greenii* Ruprecht & Türk	MK970671	-	-	-	-	-	-
T48873	5	-78.024	163.893	*Lecidella greenii* Ruprecht & Türk	MK970671	-	MN023054	*Tr*_A02	MK970698	-	-
T48874	5	-78.024	163.899	*Lecidea* UCR1	MK970676	MN023044	MN023062	*Tr*_A02	MK970697	MN023066	-
T48875	5	-78.024	163.900	*Lecidea lapicida* (Ach.) Ach. subsp.	MK970683	-	-	*Tr*_A02	MK970699	-	-
T48876a	5	-78.024	163.900	*Lecidella greenii* Ruprecht & Türk	MK970671	MN023040	-	*Tr*_A02	MK970698	-	-
T48876b	5	-78.024	163.900	*Lecidella greenii* Ruprecht & Türk	MK970671	-	MN023055	*Tr*_A02	MK970699	-	-
T48877	5	-78.024	163.900	*Carbonea vorticosa* (Flörke) Hertel	MK970656	-	MN023050	*Tr*_A02	MK970698	-	-
T48879	5	-78.057	163.747	*Lecidella greenii* Ruprecht & Türk	MK970671	-	MN023055	-	-	-	-
T48880	5	-78.058	163.740	*Lecidea polypycnidophora* Ruprecht & Türk	MK970674	-	-	-	-	-	-
T48881b	5	-78.061	163.791	*Rhizoplaca macleanii* (Dodge) Castello	MK970666	-	-	*Tr*_A02	MK970699	-	-
T48882	5	-78.057	163.817	*Lecidea polypycnidophora* Ruprecht & Türk	MK970663	-	-	*Tr*_A02	MK970699	MN023065	-
T48883b	5	-78.058	163.847	*Lecidea* sp. 5	MK620099	-	MN023063	*Tr*_A02	MK970700	-	-
T48885	5	-78.057	163.844	*Rhizoplaca macleanii* (Dodge) Castello	MK970666	-	-	*Tr*_A02	MK970698	-	-
T48887a	5	-78.114	163.854	*Lecidea cancriformis* Dodge & Baker	MK970677	-	-	*Tr*_S15	MK970692	-	-
T48887a	5	-78.070	163.711	*Lecidea cancriformis* Dodge & Baker	MK970679	MN023046	-	*Tr*_S15	MK970692	-	-
T48888b	5	-78.073	163.717	*Lecanora* cf. *mons-nivis* Darbishire	MK970658	-	-	*Tr*_A02	MK970699	-	-
T48900	5	-78.034	163.845	*Rhizoplaca macleanii* (Dodge) Castello	MK970663	-	-	*Tr*_A02	MK970698	-	-

Supplementary Table S5. Mycobiont and photobiont markers included in the study and the used primers.

Mycobiont marker	Primers
nrITS	ITS1F [8], ITS4[9], ITS1L [10], ITS4L [10]
mtSSU	mtSSU rev2 [11], mtSSU for2 mod1 (5'-AACGGCTGAACCAGCAACTTG-3'; newly designed) and mtSSU rev1 (5'-AGGYCATGATGACTTGTCTT-3', this study)
RPB1	gRPB1-A for [12], fRPB1-C rev [12], RPB1_for_Lec [10]
**Photobiont marker**	**Primers**
nrITS	18S-ITS uni-for [13], ITS4T [14], ITS1T [14], ITS4bT_mod (5'-CCAAAAGGCGTCCTGCA-3'; modified, based on Ruprecht et al. 2014 [15])
COX2	COXIIf2 [16], COXIIr [16], COXII_sense [10]
psbJ-L	psbL_for1 (5'-GTTGAATTAAATCGTACTAGT-3', this study), psbL-sense[15], psbJ-antisense [15]

Supplementary Table S6. Description of the individual steps in the creation of the phylogenies.

**Assembling, editing and aligning of the sequences**	For both symbionts, the sequences were assembled and edited using Geneious version 8.0.5 (https://www.geneious.com) and aligned with MAFFT v7.017 [17].
**Computation of the phylogenetic trees**	Maximum likelihood analyses were calculated with IQ-TREE v1.6.12 [18], using the model selection algorithm ModelFinder [19]. Branch supports were obtained with the implemented ultrafast bootstrap (UFBoot [20]; number of bootstrap alignments: 1000, maximum iteration: 1000, minimum correlation coefficient: 0.99). Additionally, a SH-aLRT branch test [21] was performed. Each branch of the resulting tree was assigned with SH-aLRT as well as UFBoot supports. The branches with SH-aLRT < 80 % and/ or UFboot < 95 % were collapsed by adding the command -minsupnew 80/95 to the script.
**Classification and labeling of the photobiont OTUs**	For the photobiont, the classification and labeling of the different operational taxonomical units (OTUs) followed the concepts of Muggia et al. [22] and Ruprecht et al. [10], using automatic barcode gap discovery (ABGD; [23]), based on the marker ITS. The threshold of 97.5 % sequence similarity set by Leavitt et al. [24] and applied by Ruprecht et al. [10] was used to ensure clear delimitation of OTUs and sub-OTUs.

Supplementary Table S7. Diversity metrics compared in this study, citations, descriptions and interpretation of each, and the used R functions.

Metric	Definition	Citations	Description and interpretation of values	R functions, R package
NRI	Net relatedness index	Webb, 2000; Webb, 2002[25,26]	Comparison of phylogenetic distances among all members of a community (pos. values = phylogenetic clustering; neg. values = phylogenetic evenness)	ses.mpd(), picante[27]
PSR	Phylogenetic species richness	Helmus, 2007[28]	PSV (phylogenetic species variability; degree to which species in a community are phylogenetically related) multiplied by species richness SR (number of species in a sample); SR after discounting species relatedness (values range from 0 = increased relatedness to SR = decreased relatedness)	psd(), picante[27]
J'	Pielou evenness index	Pielou, 1969[29]	Measure of how evenly distributed abundance is numerically among the species that exist in a community (values range from 0 = no evenness to 1 = complete evenness)	diversity(), vegan[30]
1 – J'	1 – Pielou evenness index		Values range from 0 = complete evenness to 1 = no evenness	

Supplementary Table S8. Diversity indices (left), specificity indices (middle) and BIO10, BIO12, elevation and latitude means (right) for the different mycobiont species and photobiont OTUs: *N*, number of sequences; *h*, number of haplotypes; *h / N*, ratio of *h* and *N*; *Hd*, haplotype diversity; *π*, nucleotide diversity; *NRI*, net relatedness index; *PSR*, phylogenetic species richness; *J'*, Pielou evenness index. (Note: the specificity indices were calculated for the respective symbiosis partners: *1 – J'* on the basis of species/OTUs, *NRI* and PSR on the basis of haplotypes. As a consequence, only samples where both mycobiont as well as photobiont could be identified are included.)

Mycobiont species	*N*	*h*	*h / N*	*Hd*	*π*	*NRI*	*PSR*	*1 – J'*	BIO10 mean	BIO12 mean	Elevation mean (m. a. s. l.)	Latitude mean
Carbonea sp. 2	13	3	0.231	0.564	0.0014	1.238	6.473	0.220	-7.33	120.85	518.83	-79.79
Carbonea sp. URm1	5	2	0.400	0.400	0.0128	-0.297	2.913	0.743	-6.78	138.00	524.40	-79.95
Carbonea vorticosa	11	1	0.091	-	-	1.471	2.000	1.000	-6.27	143.73	436.09	-78.08
Lecanora cf. *mons-nivis*	3	2	0.667	0.667	0.0000	1.413	2.000	1.000	-6.30	137.33	389.33	-78.07
Lecanora fuscobrunnea	31	6	0.194	0.546	0.0013	-0.423	8.332	0.399	-8.35	107.41	574.81	-81.11
Lecanora physciella	3	2	0.667	0.667	0.0014	1.473	0.011	1.000	-7.70	103.67	610.33	-84.04
Lecanora sp. 2	6	1	0.167	-	-	2.305	3.000	1.000	-6.23	141.00	606.83	-78.08
Lecanora sp. 3	7	1	0.143	-	-	3.002	2.727	1.000	-6.47	153.86	450.43	-79.14
Lecidea andersonii	8	1	0.125	-	-	-0.307	1.764	0.806	-7.19	117.88	282.50	-83.65
Lecidea cancriformis	93	18	0.194	0.797	0.0032	-1.119	10.994	0.244	-8.18	112.87	601.86	-80.82
Lecidea lapicida	1	1	1.000	-	-	-	-	1.000	-5.90	151.00	375.00	-78.02
Lecidea polypycnidophora	10	1	0.100	-	-	1.471	2.000	1.000	-6.22	141.10	411.20	-78.06
Lecidea sp. 5	1	1	1.000	-	-	-	-	1.000	-6.50	172.00	671.00	-78.06
Lecidea sp. 6	3	2	0.667	0.667	0.0106	-	-	1.000	-6.33	148.67	709.00	-78.07
Lecidea UCR1	2	1	0.500	-	-	1.450	2.000	1.000	-6.25	140.00	466.00	-78.08
Lecidella greenii	37	3	0.081	0.324	0.0007	1.933	4.600	0.929	-6.33	144.95	488.57	-78.53
Lecidella siplei	10	4	0.400	0.644	0.0012	2.974	2.916	1.000	-7.52	136.80	322.30	-84.21
Lecidella sp. nov2	9	3	0.333	0.556	0.0073	1.643	0.403	0.821	-7.90	100.33	787.67	-83.87
Rhizoplaca macleanii	51	8	0.157	0.707	0.0019	4.209	4.860	1.000	-6.18	149.92	684.53	-78.07

Photobiont OTU	*N*	*h*	*h / N*	*Hd*	*π*	*NRI*	*PSR*	*1 – J'*	BIO10 mean	BIO12 mean	Elevation mean (m. a. s. l.)	Latitude mean
*Tr*_A02	165	14	0.085	0.702	0.0025	1.739	16.265	0.220	-6.47	144.24	538.61	-79.20
*Tr*_A04a	3	3	1.000	1.000	0.0023	-0.759	3.000	0.784	-8.70	116.67	491.00	-82.46
*Tr*_I01	10	6	0.600	0.844	0.0084	0.036	1.955	0.695	-8.12	98.60	555.70	-81.19
*Tr*_I17	2	1	0.500	-	-	-	-	1.000	-7.60	101.50	358.50	-82.21
*Tr*_S02	58	8	0.138	0.573	0.0049	0.538	7.970	0.580	-7.89	120.25	565.10	-82.12
*Tr*_S15	10	1	0.100	-	-	0.578	2.596	0.728	-6.16	139.10	689.10	-78.12
*Tr*_S18	32	1	0.031	-	-	2.529	3.431	0.828	-9.91	77.88	696.25	-80.02

Supplementary Table S9. Network matrix giving the number of associations between the mycobiont species and photobiont OTUs.

	Photobiont OTU						
**Mycobiont species**	*Tr*_A02	*Tr*_A04a	*Tr*_I01	*Tr*_I17	*Tr*_S02	*Tr*_S15	*Tr*_S18
Carbonea sp. 2	4	2	-	-	3	2	1
Carbonea sp. URm1	4	-	1	-	-	-	-
Carbonea vorticosa	9	-	-	-	-	-	-
Lecanora cf. *mons-nivis*	3	-	-	-	-	-	-
Lecanora fuscobrunnea	11	-	3	-	11	-	2
Lecanora physciella	-	-	-	-	2	-	-
Lecanora sp. 2	6	-	-	-	-	-	-
Lecanora sp. 3	7	-	-	-	-	-	-
Lecidea andersonii	7	-	-	-	1	-	-
Lecidea cancriformis	7	1	6	2	34	7	28
Lecidea lapicida	1	-	-	-	-	-	-
Lecidea polypycnidophora	9	-	-	-	-	-	-
Lecidea sp. 5	1	-	-	-	-	-	-
Lecidea sp. 6	3	-	-	-	-	-	-
Lecidea UCR1	2	-	-	-	-	-	-
Lecidella greenii	31	-	-	-	-	1	-
Lecidella siplei	9	-	-	-	-	-	-
Lecidella sp. nov2	-	-	-	-	8	-	1
Rhizoplaca macleanii	51	-	-	-	-	-	-

References

1 Gunn, B. M. & Walcott, R. I. The geology of the Mt Markham region, Ross dependency, Antarctica. New Zealand Journal of Geology and Geophysics 5, 407-426 (1962).

2 Simpson, A. L. & Cooper, A. F. Geochemistry of the Darwin Glacier region granitoids, southern Victoria Land. Antarctic Science 14, 425-426, doi:Doi 10.1017/S0954102002000226 (2002).

3 Doran, P. T. et al. Valley floor climate observations from the McMurdo Dry Valleys, Antarctica, 1986–2000. Journal of Geophysical Research: Atmospheres 107, ACL 13-11-ACL 13-12 (2002).

4 Stichbury, G., Brabyn, L., Allan Green, T. & Cary, C. Spatial modelling of wetness for the Antarctic Dry Valleys. Polar Research 30, 6330 (2011).

5 Yung, C. C. M. et al. Characterization of Chasmoendolithic Community in Miers Valley, McMurdo Dry Valleys, Antarctica. Microbial Ecology 68, 351-359 (2014).

6 Perez-Ortega, S., Ortiz-Alvarez, R., Allan Green, T. G. & de Los Rios, A. Lichen myco- and photobiont diversity and their relationships at the edge of life (McMurdo Dry Valleys, Antarctica). FEMS Microbiol Ecol 82, 429-448, doi:10.1111/j.1574-6941.2012.01422.x (2012).

7 Wagner, M. et al. Myco- and photobiont associations in crustose lichens in the McMurdo Dry Valleys (Antarctica) reveal high differentiation along an elevational gradient. Polar Biology 43, 1967-1983, doi:10.1007/s00300-020-02754-8 (2020).

8 Gardes, M. & Bruns, T. D. Its Primers with Enhanced Specificity for Basidiomycetes - Application to the Identification of Mycorrhizae and Rusts. Molecular Ecology 2, 113-118 (1993).

9 White, T. J. et al. PCR Protocols: A Guide to Methods and Applications. (Academic Press, 1990).

10 Ruprecht, U., Fernandez-Mendoza, F., Turk, R. & Fryday, A. M. High levels of endemism and local differentiation in the fungal and algal symbionts of saxicolous lecideoid lichens along a latitudinal gradient in southern South America. Lichenologist (Lond) 52, 287-303, doi:10.1017/S0024282920000225 (2020).

11 Ruprecht, U., Lumbsch, H. T., Brunauer, G., Green, T. G. A. & Türk, R. Diversity of Lecidea (Lecideaceae, Ascomycota) species revealed by molecular data and morphological characters. Antarctic Science 22, 727-741, doi:Doi 10.1017/S0954102010000477 (2010).

12 Matheny, P. B., Liu, Y. J. J., Ammirati, J. F. & Hall, B. D. Using RPB1 sequences to improve phylogenetic inference among mushrooms (Inocybe, Agaricales). American Journal of Botany 89, 688-698 (2002).

13 Ruprecht, U., Brunauer, G. & Printzen, C. Genetic diversity of photobionts in Antarctic lecideoid lichens from an ecological viewpoint. Lichenologist 44, 661-678, doi:Doi 10.1017/S0024282912000291 (2012).

14 Kroken, S. & Taylor, J. W. Phylogenetic species, reproductive mode, and specificity of the green alga Trebouxia forming lichens with the fungal genus Letharia. Bryologist 103, 645-660 (2000).

15 Ruprecht, U., Brunauer, G. & Turk, R. High photobiont diversity in the common European soil crust lichen Psora decipiens. Biodiversity and Conservation 23, 1771-1785, doi:DOI 10.1007/s10531-014-0662-1 (2014).

16 Lindgren, H. et al. High fungal selectivity for algal symbionts in the genus Bryoria. Lichenologist 46, 681-695, doi:10.1017/S0024282914000279 (2014).

17 Katoh, K., Misawa, K., Kuma, K. & Miyata, T. MAFFT: a novel method for rapid multiple sequence alignment based on fast Fourier transform. Nucleic Acids Research 30, 3059-3066, doi:DOI 10.1093/nar/gkf436 (2002).

18 Nguyen, L.-T., Schmidt, H. A., von Haeseler, A. & Minh, B. Q. IQ-TREE: A Fast and Effective Stochastic Algorithm for Estimating Maximum-Likelihood Phylogenies. Molecular Biology and Evolution 32, 268-274, doi:10.1093/molbev/msu300 (2014).

19 Kalyaanamoorthy, S., Minh, B. Q., Wong, T. K. F., von Haeseler, A. & Jermiin, L. S. ModelFinder: fast model selection for accurate phylogenetic estimates. Nat Methods 14, 587-589, doi:10.1038/nmeth.4285 (2017).

20 Minh, B. Q., Nguyen, M. A. T. & von Haeseler, A. Ultrafast Approximation for Phylogenetic Bootstrap. Molecular Biology and Evolution 30, 1188-1195, doi:10.1093/molbev/mst024 (2013).

21 Guindon, S. et al. New Algorithms and Methods to Estimate Maximum-Likelihood Phylogenies: Assessing the Performance of PhyML 3.0. Syst Biol 59, 307-321, doi:10.1093/sysbio/syq010 (2010).

22 Muggia, L. et al. Formally described species woefully underrepresent phylogenetic diversity in the common lichen photobiont genus Trebouxia (Trebouxiophyceae, Chlorophyta): An impetus for developing an integrated taxonomy. Molecular Phylogenetics and Evolution 149, 106821, doi:10.1016/j.ympev.2020.106821 (2020).

23 Puillandre, N., Lambert, A., Brouillet, S. & Achaz, G. ABGD, Automatic Barcode Gap Discovery for primary species delimitation. Mol Ecol 21, 1864-1877, doi:10.1111/j.1365-294X.2011.05239.x (2012).

24 Leavitt, S. D. et al. Fungal specificity and selectivity for algae play a major role in determining lichen partnerships across diverse ecogeographic regions in the lichen-forming family Parmeliaceae (Ascomycota). Mol Ecol 24, 3779-3797, doi:10.1111/mec.13271 (2015).

25 Webb, C. O. Exploring the phylogenetic structure of ecological communities: An example for rain forest trees. American Naturalist 156, 145-155, doi:Doi 10.1086/303378 (2000).

26 Webb, C. O., Ackerly, D. D., McPeek, M. A. & Donoghue, M. J. Phylogenies and community ecology. Annual Review of Ecology and Systematics 33, 475-505, doi:10.1146/annurev.ecolysis.33.010802.150448 (2002).

27 Kembel, S. W. et al. Picante: R tools for integrating phylogenies and ecology. Bioinformatics 26, 1463-1464 (2010).

28 Helmus, M. R., Bland, T. J., Williams, C. K. & Ives, A. R. Phylogenetic measures of biodiversity. American Naturalist 169, E68-E83, doi:Doi 10.1086/511334 (2007).

29 Pielou, E. C. An Introduction to Mathematical Ecology. (Wiley, 1969).

30 Oksanen, J. et al. vegan: Community Ecology Package. R package version 2.5–6. (2019).
