# Supplementary material for: Macroclimatic conditions as main drivers for symbiotic association patterns in lecideoid lichens along the Transantarctic Mountains, Ross Sea region, Antarctica
Source: Sci Rep. 2021 Dec 6;11:23460. doi: 10.1038/s41598-021-02940-6 (PMC8648759; doi:10.1038/s41598-021-02940-6)
Supplement: Supplementary file 2 — Supplementary Information 2. [file 41598_2021_2940_MOESM2_ESM.pdf]

Supplementary Material 2: Figures

Contents

Page

Supplementary Figure S1 ______ 2
Supplementary Figure S2 ______ 3
Supplementary Figure S3 ______ 4
Supplementary Figure S4 ______ 5
Supplementary Figure S5 ______ 6
Supplementary Figure S6 ______ 7
Supplementary Figure S7 ______ 7
Supplementary Figure S8 ______ 8
Supplementary Figure S9 ______ 8
Supplementary Figure S10 ______ 9
Supplementary Figure S11 ______ 9
References ______ 9

Supplementary Figure S1. Phylogeny of mycobiont specimen based on multi-locus sequence data (nrITS, mtSSU and RPB1; calculated with IQ-TREE[1]; branches with SH-aLRT < 80 % and UFboot < 95 % were collapsed).

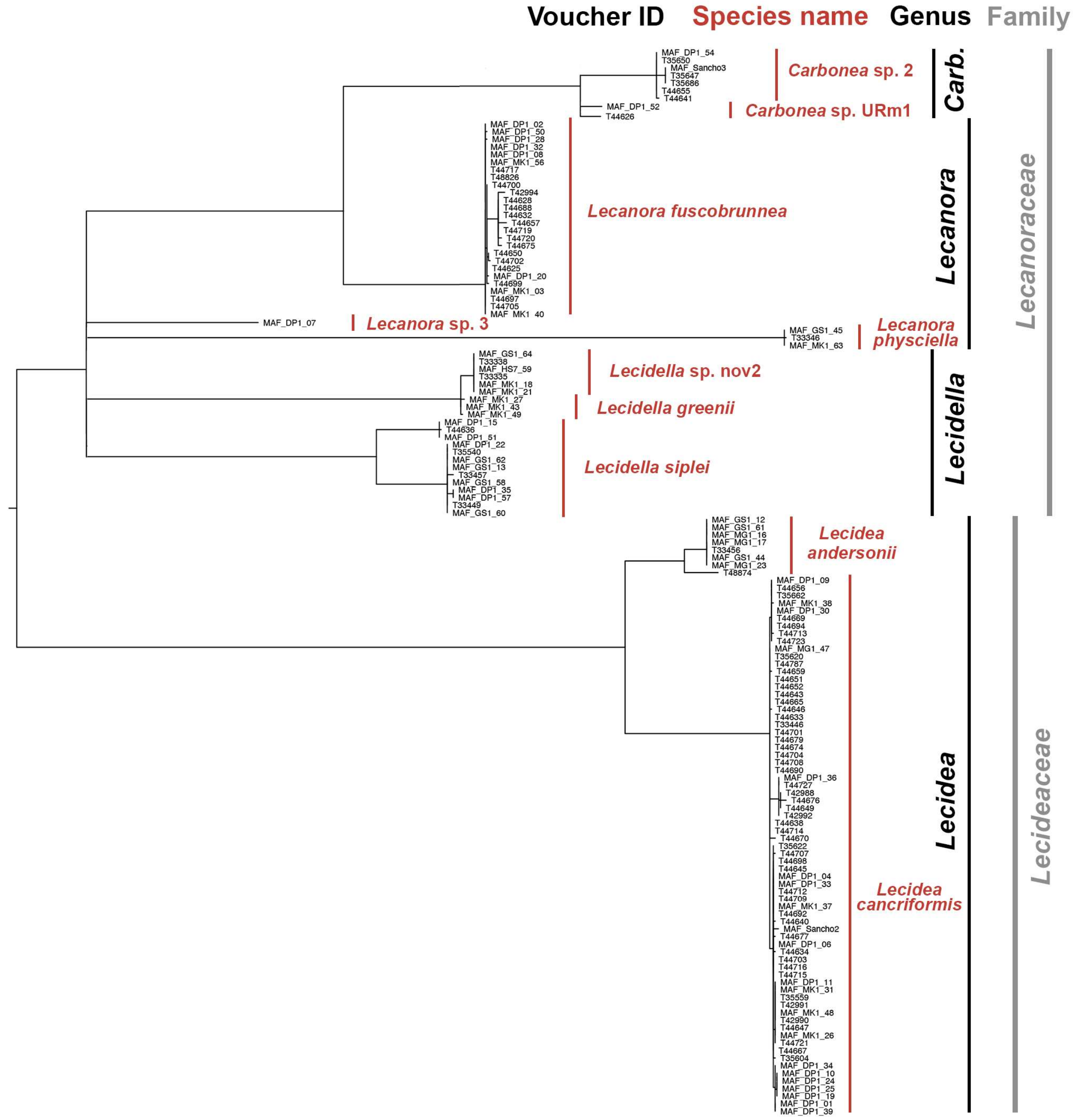

Supplementary Figure S2. Phylogeny of all mycobiont specimen based on the marker nrITS (calculated with IQ-TREE[1]; branches with SH-aLRT < 80 % and UFboot < 95 % were collapsed).

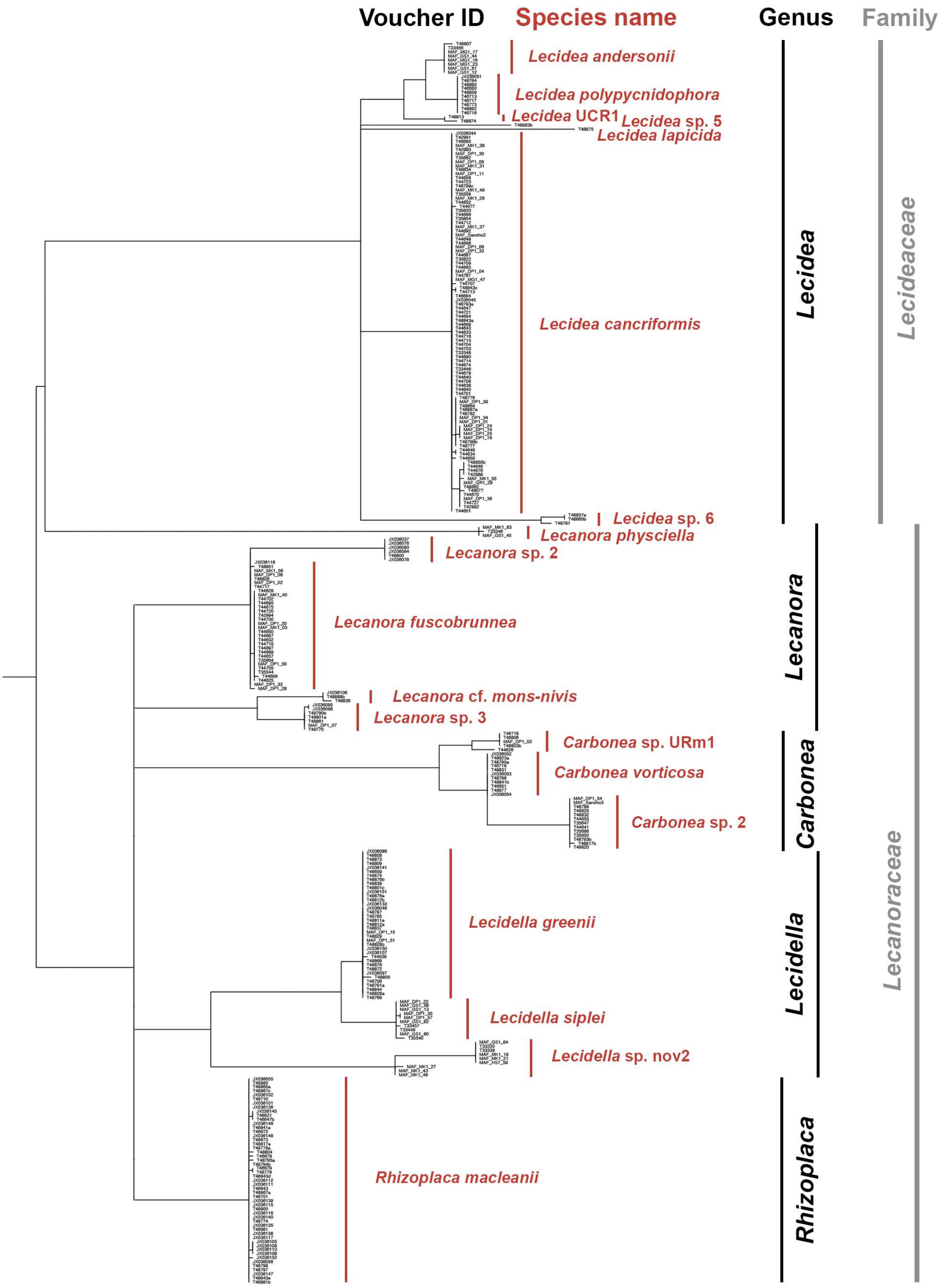

Supplementary Figure S3. Phylogeny of photobiont specimen based on multi-locus sequence data (nrITS, psbJ-L and COX2; calculated with IQ-TREE[1]; branches with SH-aLRT < 80 % and UFboot < 95 % were collapsed).

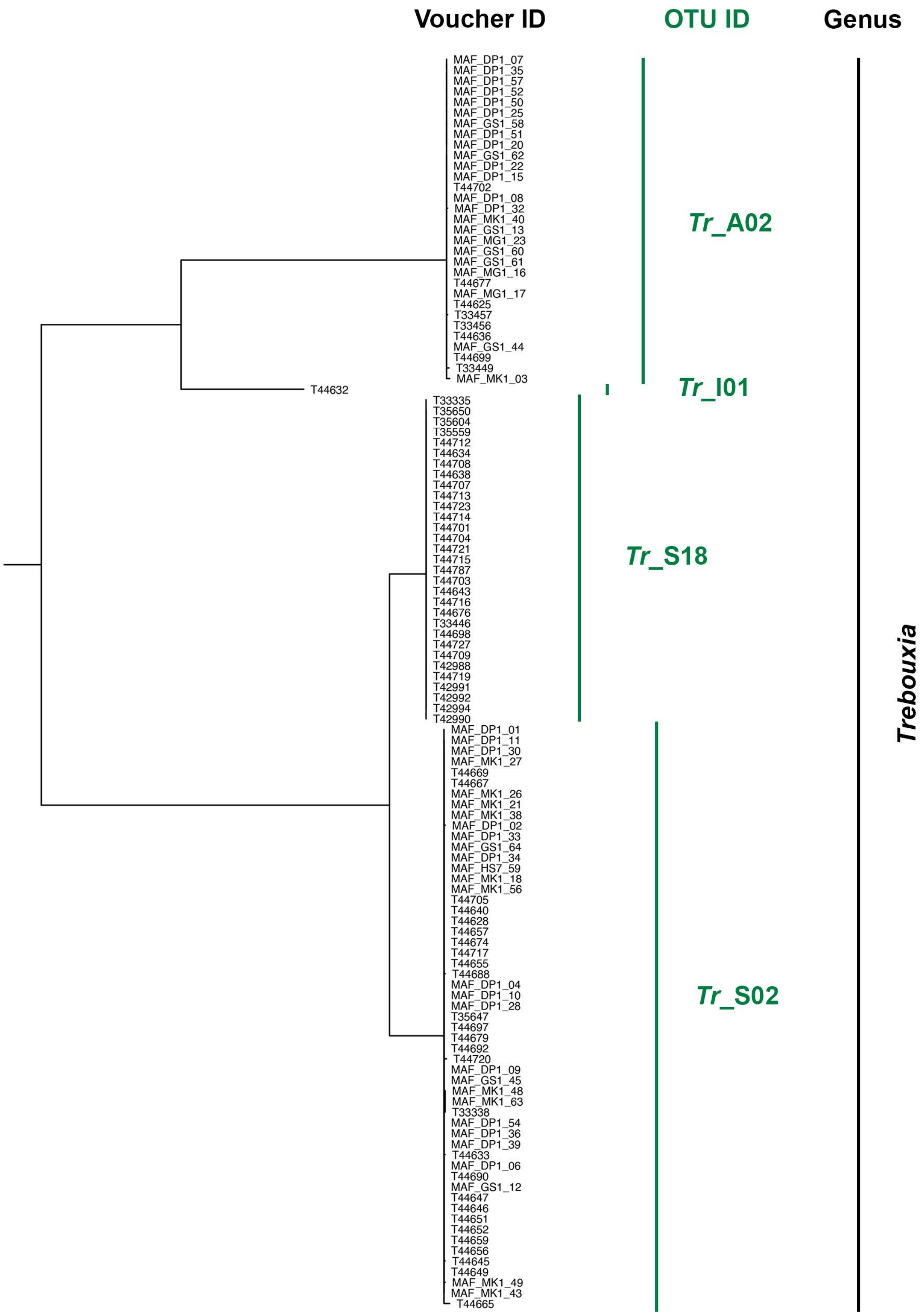

Supplementary Figure S4. Phylogeny of all photobiont specimen based on the marker nrITS (calculated with IQ-TREE[1]; branches with SH-aLRT < 80 % and UFboot < 95 % were collapsed).

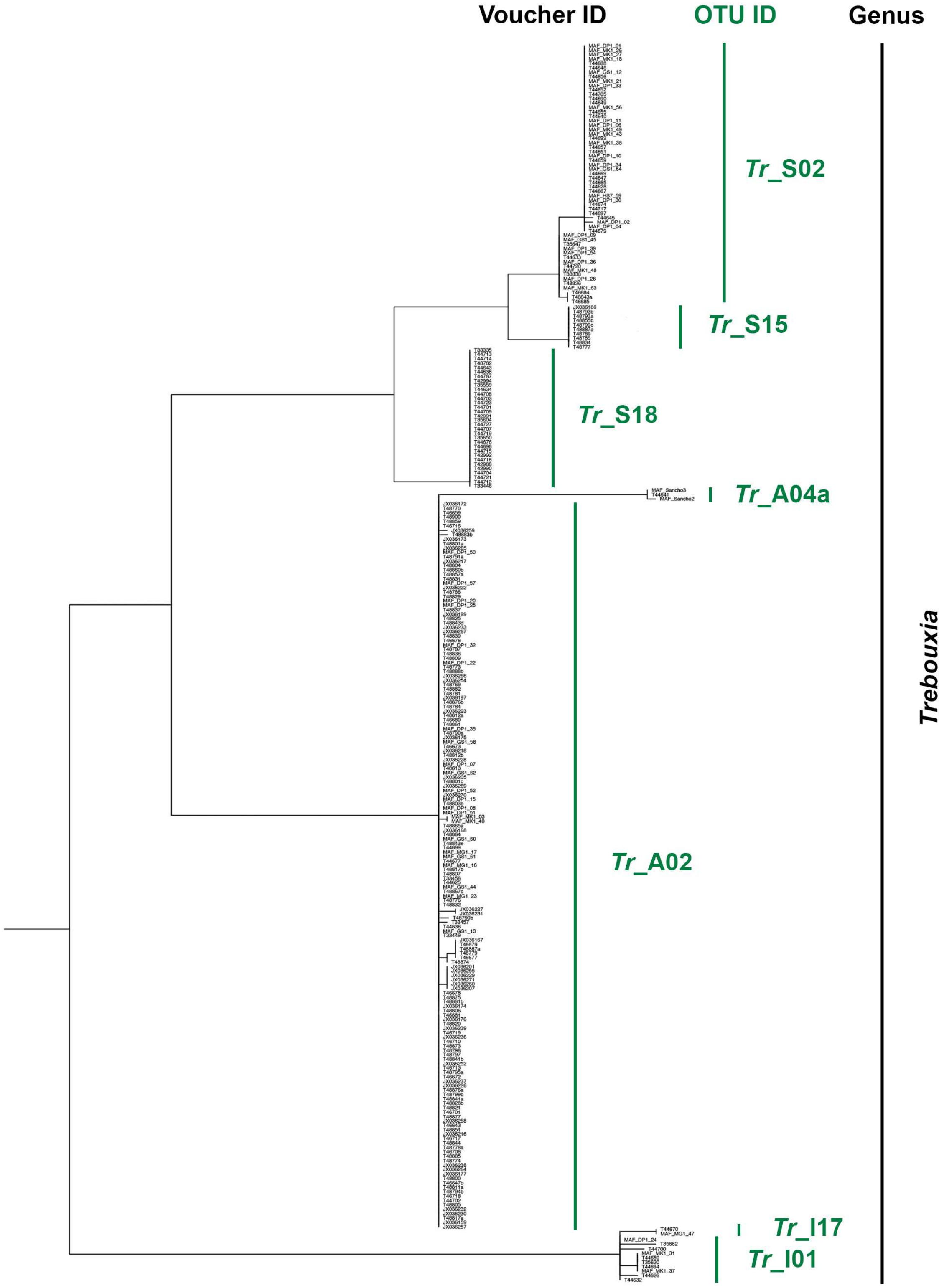

Supplementary Figure S5. Barplots giving the number of samples per mycobiont species/ photobiont OTU and area included in this study. (a) Mycobiont species (total sample size: n = 306), (b) photobiont OTUs (total sample size: n = 281).

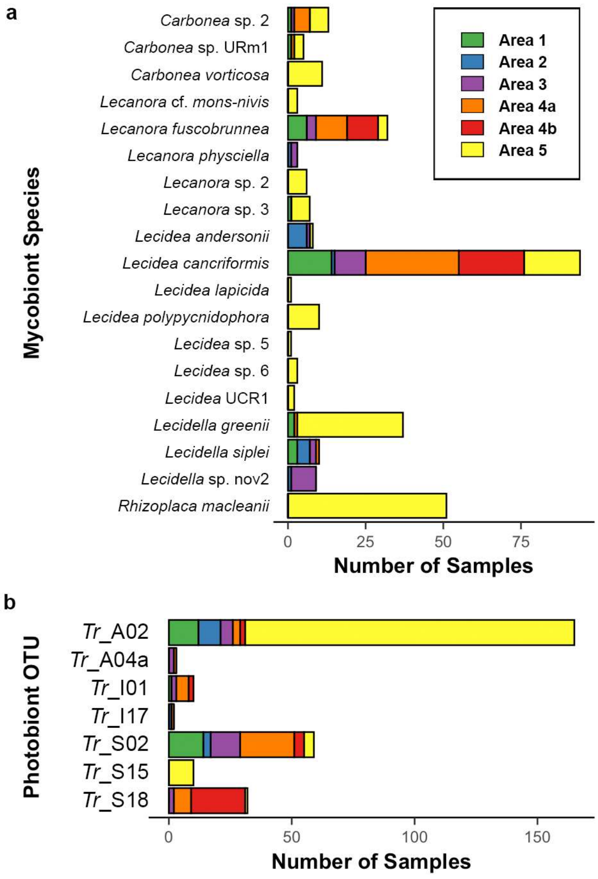

Supplementary Figure S6. Rarefaction/extrapolation curves. Based on sample coverage, with 95% confidence intervals (shaded areas) comparing alpha diversity of a, mycobiont species, and b, photobiont OTUs, within the different areas.

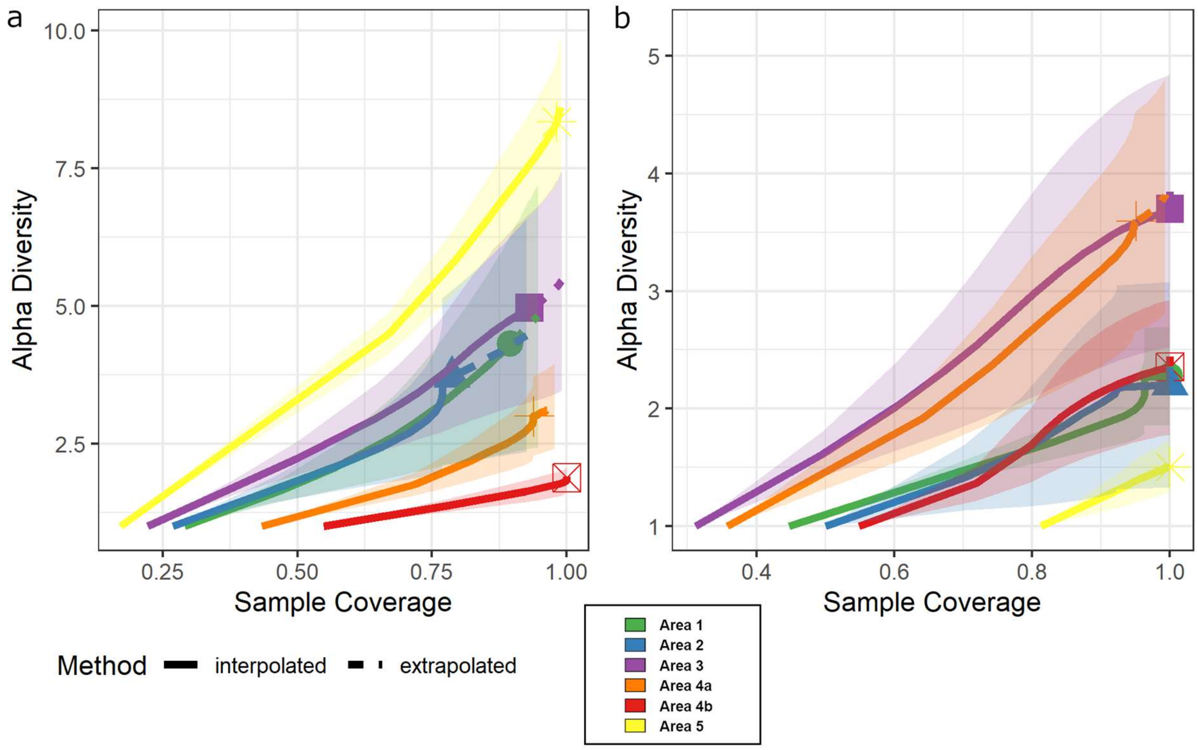

Supplementary Figure S7. Correlation plot. Percentage of *Trebouxia* OTU A02 samples against mean values of BIO10 (mean temperature of warmest quarter) for the different areas.

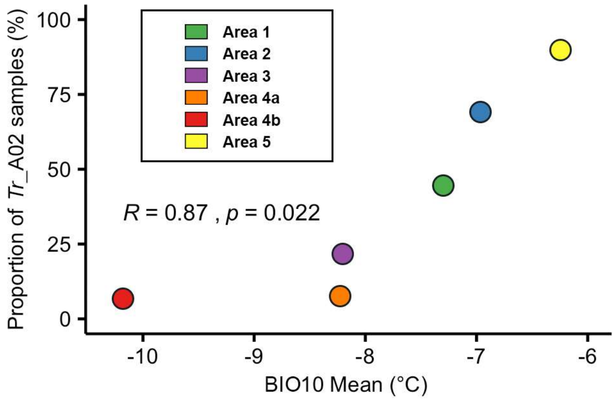

Supplementary Figure S8. Correlation plot. Alpha diversity values of mycobiont species against BIO10 (mean temperature of warmest quarter) mean values of the different areas.

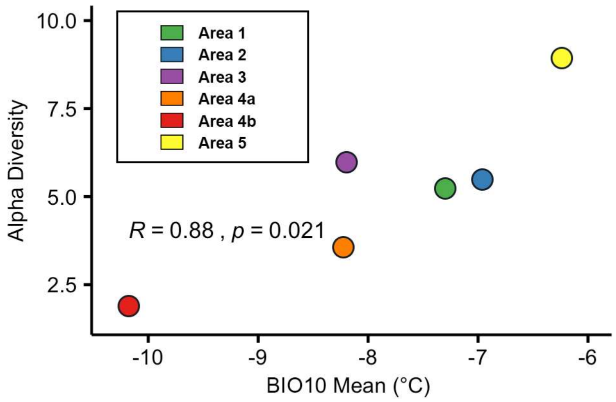

Supplementary Figure S9. Ordination plots showing the similarity of mycobiont samples with n ≥ 10 after constrained analysis of principal coordinates. Samples located closer to each other are also more similar in terms of the environmental factors elevation, BIO10 and BIO12. The first constrained axis CAP1 explained 13.77 % of the variance, the second constrained axis CAP2 1.76 % of the variance. Only the first axis was significant (F = 17.1640, p = 0.001).

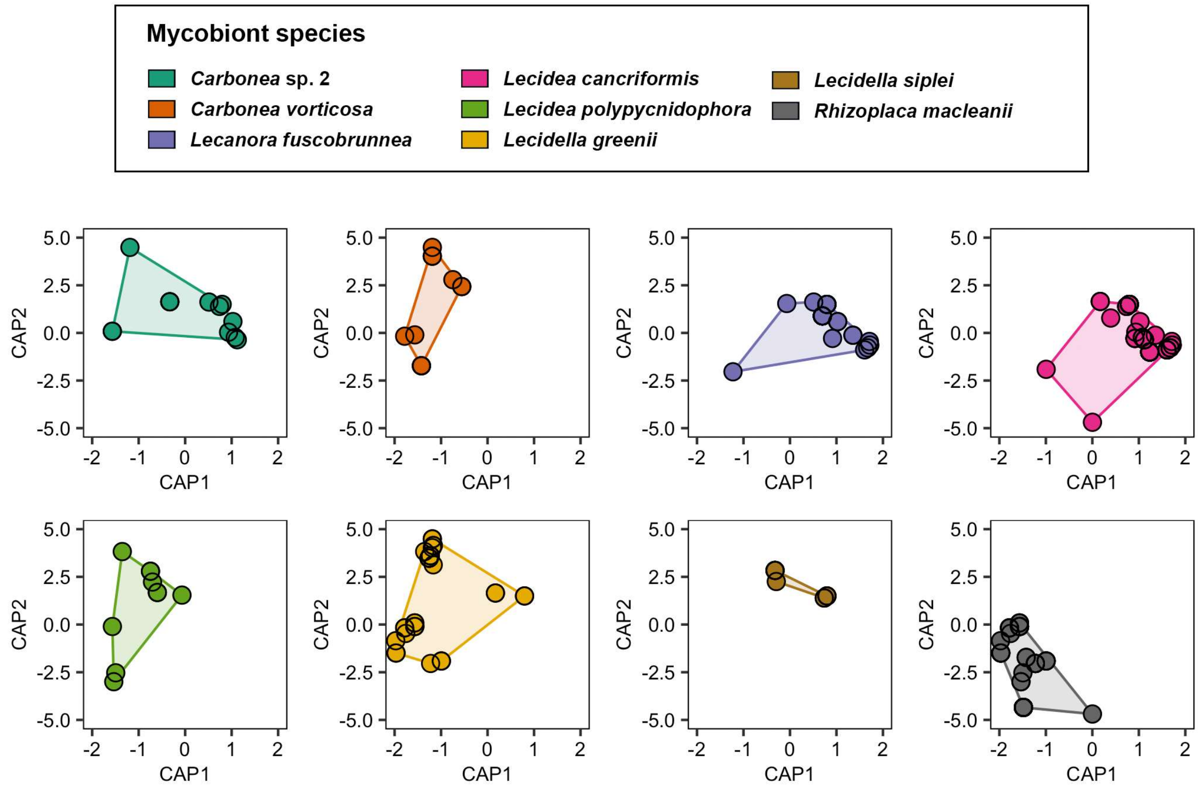

Supplementary Figure S10. Ordination plots showing the similarity of photobiont OTUS with n ≥ 10 after constrained analysis of principal coordinates. Samples located closer to each other are also more similar in terms of the environmental factors elevation, BIO10 and BIO12. The first constrained axis CAP1 explained 36.87 % of the variance, the second constrained axis CAP2 1.46 % of the variance. Only the first axis was significant ($F = 57.0275$, $p = 0.001$).

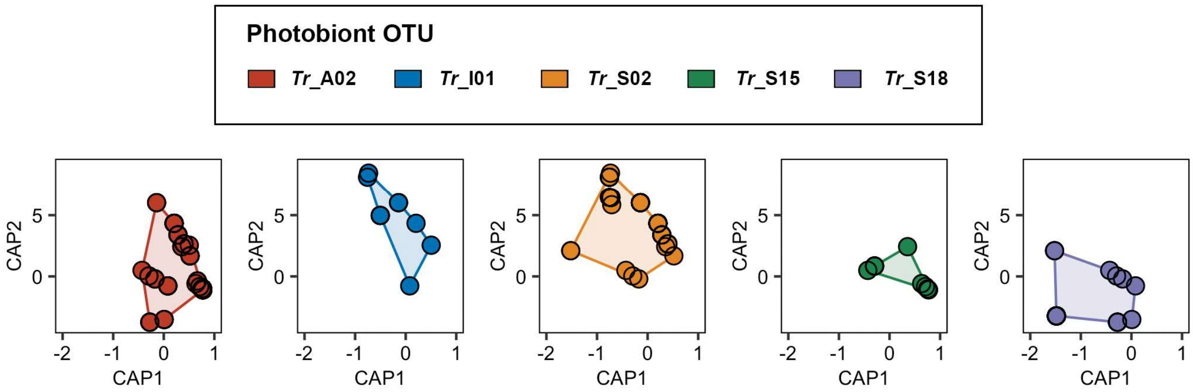

Supplementary Figure S11. Haplotype networks based on multi-locus sequence data, showing the spatial distribution within the different areas. (a) *Lecidea cancriformis*, (b) *Trebouxia* OTU S02. Roman numerals at the center of the pie charts refer to the haplotype IDs based on ITS data (cf., Fig. 2 and Fig. 3 of main text). The italic numbers next to the pie charts give the total number of samples per haplotype. The circle sizes reflect relative frequency within the species; the frequencies were clustered in ten (e.g. the circles of all haplotypes making up between 20-30 % have the same size).

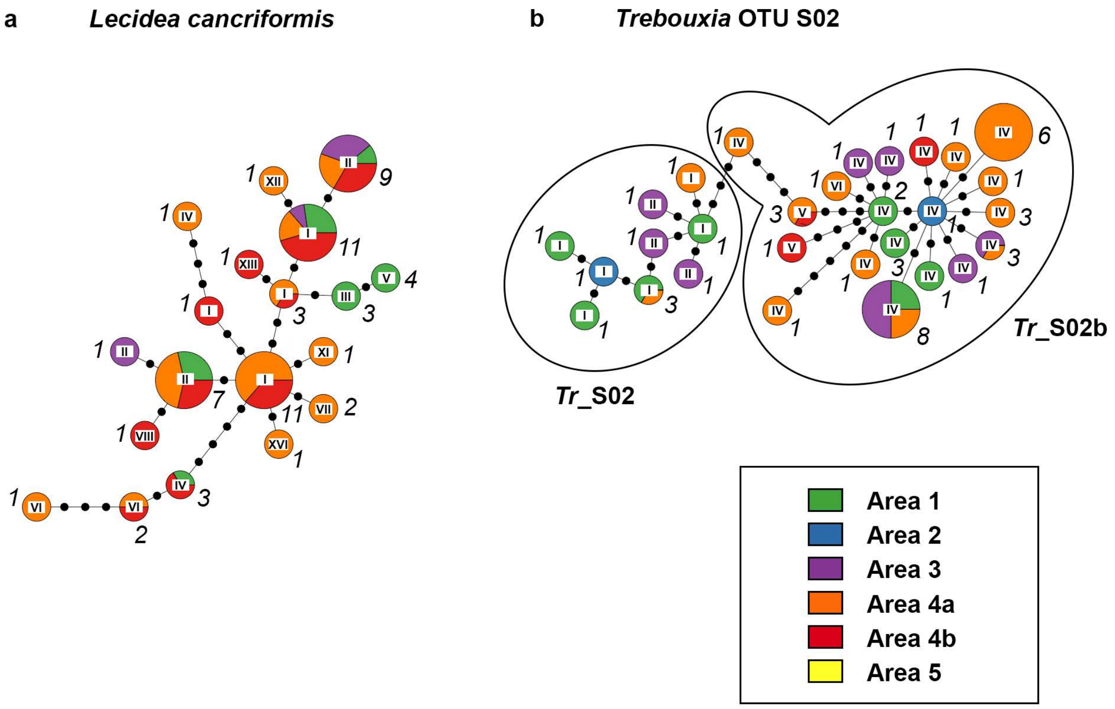

References

1 Nguyen, L.-T., Schmidt, H. A., von Haeseler, A. & Minh, B. Q. IQ-TREE: a fast and effective stochastic algorithm for estimating maximum-likelihood phylogenies. *Mol Biol Evol* **32**, 268-274, doi:10.1093/molbev/msu300 (2014).
